# Model-based estimates of transmission of respiratory syncytial virus within households

**DOI:** 10.1016/j.epidem.2018.12.001

**Published:** 2019-06

**Authors:** Ivy K. Kombe, Patrick K. Munywoki, Marc Baguelin, D. James Nokes, Graham F. Medley

**Affiliations:** a KEMRI-Wellcome Trust Research Programme, KEMRI Center for Geographical Medical Research-Coast, P.O. Box 230-80108, Kilifi, Kenya; b Centre for Mathematical Modelling of Infectious Disease and Department of Global Health and Development, London School of Hygiene and Tropical Medicine, London, WC1H 9SH, UK; c Centre for Mathematical Modelling of Infectious Disease and Department of Infectious Disease Epidemiology, London School of Hygiene and Tropical Medicine, London, WC1H 9SH, UK; d School of Life Sciences and Zeeman Institute for Systems Biology & Infectious Disease Epidemiology Research, University of Warwick, Coventry, CV4 7AL, UK

**Keywords:** Respiratory syncytial virus, Household, Transmission

## Abstract

**Introduction:**

Respiratory syncytial virus (RSV) causes a significant respiratory disease burden in the under 5 population. The transmission pathway to young children is not fully quantified in low-income settings, and this information is required to design interventions.

**Methods:**

We used an individual level transmission model to infer transmission parameters using data collected from 493 individuals distributed across 47 households over a period of 6 months spanning the 2009/2010 RSV season. A total of 208 episodes of RSV were observed from 179 individuals. We model competing transmission risk from within household exposure and community exposure while making a distinction between RSV groups A and B.

**Results:**

We find that 32–53% of all RSV transmissions are between members of the same household; the rate of pair-wise transmission is 58% (95% CrI: 30–74%) lower in larger households (≥8 occupants) than smaller households; symptomatic individuals are 2–7 times more infectious than asymptomatic individuals i.e. 2.48 (95% CrI: 1.22–5.57) among symptomatic individuals with low viral load and 6.7(95% CrI: 2.56–16) among symptomatic individuals with high viral load; previous infection reduces susceptibility to re-infection within the same epidemic by 47% (95% CrI: 17%–68%) for homologous RSV group and 39% (95%CrI: -8%-69%) for heterologous group; RSV B is more frequently introduced into the household, and RSV A is more rapidly transmitted once in the household.

**Discussion:**

Our analysis presents the first transmission modelling of cohort data for RSV and we find that it is important to consider the household social structuring and household size when modelling transmission. The increased infectiousness of symptomatic individuals implies that a vaccine against RSV related disease would also have an impact on infection transmission. Together, the weak cross immunity between RSV groups and the possibility of different transmission niches could form part of the explanation for the group co-existence.

## Graphical abstract

**Figure:**
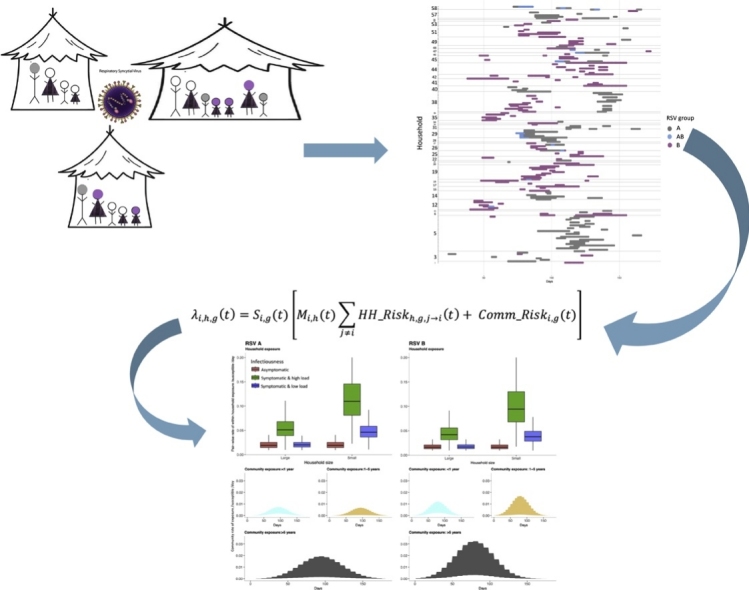

## 1 Introduction

Respiratory syncytial virus (RSV) is an ubiquitous RNA virus infection that is a major cause of lower respiratory tract disease in children under 5 years of age worldwide (Nair et al., 2010; Shi et al., 2015). The estimated global burden of RSV associated acute lower respiratory tract infection (ALRI) in 2015 in under 5 year olds is 33.0 million (21.6–50.3), most of which occurs in developing countries (30.5 million) (Shi et al., 2017). Of the 3.2 (2.7–3.8) million hospital admissions associated with RSV in the under 5 s, 1.4 (1.2–1.7) million occurred in the 0–5 months age group, and 1.2 (1.0–1.5) million occurred in developing countries.

Despite 50 years of vaccine research none is yet licensed for the prevention of RSV infection or disease. There are currently over fifty vaccines in different stages of development: many with the aim of prevention of early infant RSV disease. While the most advanced (in phase III trials) is a maternal vaccine to boost transplacental antibody transfer (Nigel Thomas (2017); “RSV Vaccine Snapshot (2016); WHO (2016), a variety of product types and range of strategies for protecting young children are under investigation including indirect protection by targeting older infants, elder siblings and family cocooning (Anderson et al., 2013; Kinyanjui et al., 2015; Poletti et al., 2015).

Prior to vaccine introduction, drivers of transmission need to be well understood in order to predict the potential public health impact of implementation. Investigating outbreaks within the household setting could help to further characterize RSV transmission. The household is an important unit of study for diseases that are transmitted through close contact. The quantitative analysis of household outbreaks has been conducted for influenza (Cauchemez et al., 2009, 2004; House et al., 2012; Klick et al., 2011; Lau et al., 2015; Wu et al., 2006; Yang et al., 2015). This has led to quantification of transmissibility within the household, improved understanding of the factors that determine level of transmission such as household size and effectiveness of different household level interventions (Tsang et al., 2016). To date studies of RSV transmission within households or families have been largely observational. One of the earliest is a household cohort study in the USA in which 36 families were followed up for 2 months during the 1974/1975 RSV season (Hall et al., 1976). This study found that RSV attack rates in households were high, more so in infants. Older siblings to infants were found to be the most likely index cases in household outbreaks, and illness was found to have an age-related severity. Several other studies over the years across different settings have highlighted the importance of older children in household outbreaks (Heikkinen et al., 2015; Jacoby and Glass, 2017; Munywoki et al., 2014) which could have implications for control strategies (Graham, 2014).

In Kenya, a household cohort study conducted in a rural coastal community during the 2009/2010 RSV epidemic has revealed several patterns. In addition to the importance of older children (Munywoki et al., 2014), bigger household size and infection with RSV group B, among other factors, were found to be independently associated with increased risk of asymptomatic infection (Munywoki et al., 2015a); shedding duration estimates (using molecular diagnostics) were 11.2 days on average, and longer than the previous range reported of 3.9–7.4 days (Munywoki et al., 2015b); individuals experiencing the first infection of an RSV season were found to shed more virus relative to secondary infections; children under 1 year old, symptomatic shedders and RSV A and B co-infected individuals were identified as the most likely to transmit due to their relatively higher viral loads (Wathuo et al., 2016).

RSV can be categorized into two antigenically and genetically distinct groups, RSV A and RSV B (Cane, 2001). These groups, thought to have diverged about 350 years ago (Zlateva et al., 2005), have been observed to co-exist geographically and temporally with most outbreaks being dominated by RSV A and, in some locations, clear patterns of alternating dominance (White et al., 2005). Within the RSV groups are subgroups or genotypes whose frequency changes from season to season, with some genotypes undergoing complete replacement over time (Agoti et al., 2013, 2012; Park et al., 2017; Rodriguez-Fernandez et al., 2017; Song et al., 2017; Thongpan et al., 2017). This pattern of group and genotype replacement is thought to be due to a herd immunity effect (Botosso et al., 2009; Cane, 2001; Pretorius et al., 2013; White et al., 2005). A phylogenetic analysis of RSV A sequences from the Kenyan household study showed that most infections arise from a single variant introduction followed by accumulation of household specific variation, i.e. cases arise more from within household spread rather than multiple introductions (Agoti et al., 2017).

However, there is yet to be a mechanistic analysis of RSV household outbreak data that consolidates information on the characteristics of infection episodes and characteristics of the host population into a single dynamic framework. Inference could then be drawn on the competing risks of within household exposure and community (external to household) exposure, in order to quantify the importance of households in RSV transmission. We proposed to use an individual-based approach within a Bayesian framework to analyze the household cohort data from Kenya to further understand transmission dynamics. We also explore the differences and interactions between RSV groups.

## 2 Material and methods

### 2.1 Data

The data to be used were collected from a household cohort study conducted in rural coastal Kenya within the Kilifi Health and Demographic Surveillance System (KHDSS) during the 2009/2010 RSV epidemic. Details of the study have been published elsewhere (Munywoki, 2013; Munywoki et al., 2015a, 2015b, 2014). In brief, the infant-centric study recruited household members using the criteria that the infant was born after 1 April 2009 (after the previous RSV epidemic) and had at least 1 older sibling less than 13 years old. Deep nasopharyngeal swab (NPS) samples were collected every 3–4 days regardless of symptoms, together with a record of clinical illness. The samples were tested for RSV antigen using an in-house real-time multiplex polymerase chain reaction (PCR) assay. A sample was considered antigen positive if the PCR cycle threshold (Ct) value was 35.0 or below. Positive Ct values were then converted to viral load (log_10_ RNA equivalent). A household was defined as a group of individuals living in the same compound and eat together. The data contain information from 493 individuals spread across 47 households whose dates of data collection span 180 days. The household sizes range from 4 to 37 occupants with a median of 8 members.

An RSV A/B shedding episode is defined as a period within which an individual provided PCR positive samples for RSV A/B that were no more than 14 days apart. A shedding episode is referred as symptomatic if within the window of virus shedding, there is at least one day where symptoms were recorded. The symptoms of interest are those of an acute respiratory illness (ARI), which are: cough, or nasal discharge/blockage, or difficulty breathing. Sampling of the study population was done in 3–4 day intervals, as such, complete duration of shedding and ARI episodes had to be imputed, and missing viral loads were linearly interpolated. Shedding durations were imputed first, after which, if there were any days of recorded ARI within shedding episodes, the total duration of the ARI was imputed based on the days of recorded symptoms. As such, the length of an ARI episode within a shedding episode can be ≤ length of related shedding episode.

Details of the imputation of episodes and interpolation of viral load can be found in the Supplementary appendix section.

We categorized days of shedding according to viral load and symptoms into 4 categories to compare infectiousness: low viral load and asymptomatic, high viral load and asymptomatic, low viral load and symptomatic and, high viral load and symptomatic. High viral load is defined as >6 log_10_ viral copy number (or a PCR Ct value <23.05).

### 2.2 Transmission model

We built a mechanistic model for RSV that tracks infection onset at the individual host level. The main assumptions about transmission are contained in the equation giving the per capita rate of exposure (to infection) per unit time, also known as the infection hazard. The rate of exposure to a particular RSV group (index *g*) is given for a particular individual, (index *i*) from a given household (index *h*) at a given day (index *t*) and is specified by the notation $\lambda_{i,h,g}\left(t\right)$. We assume that an individual can be exposed to infection in the household they occupy and from external infection sources and as such, decompose the rate of exposure into two parts, a within household component and a community component.

#### 2.2.1 Within household exposure

For an individual *i*, in household *h*, the rate of exposure at a given time *t*, is a summation of rates from all the infectious individuals in their household. The rate of exposure from a single infectious housemate (index *j*) is assumed to depend on the size of the household and the viral load and symptom status. We consider the household size effect as a binary variable where a house with >8 members is considered large. We consider viral load and symptom status as one variable with 4 categories: low viral load and no symptoms, high viral load and no symptoms, low viral load and symptomatic, high viral load and symptomatic. The household rate of exposure from individual *j* to *i* is thus give as:

$${HH\_Risk}_{h,g,j\to i}\left(t\right)=\;{\eta_{g}\;\times \;\psi }_{H}\left({Household\_size}_{i}\right)\;\times \;\psi_{I,inf}\left({Infectivity}_{j,h,g}(t)\right)$$

$\eta_{g}$ is the baseline rate of exposure in the household which is estimated for each of the two RSV groups, RSV A and RSV B. $\psi_{H}$ is the coefficient modifying exposure in large household relative to small households and $\psi_{I,inf}$ is the coefficient modifying infectiousness based on viral load and symptom status. The within household rate of exposure only affects susceptible individuals who are present in the household, as such this rate is multiplied by a binary variable $M_{i,h}(t)$ = 0 if *i* is not present in the household at time *t* and $M_{i,h}(t)$ = 1 if *i* is present.

#### 2.2.2 Community exposure

For a susceptible individual *i*, this external to the household source of exposure is assumed to represent both sampled and unsampled cases from other households. Community exposure is assumed to depend on the age of the susceptible individual and time. Age is treated as a categorical variable. The community rate of exposure is thus given as:

$${Comm_{Risk}}_{i,g}\left(t\right)=\varepsilon_{g}\;\times \;f_{g}\left(t\right)\;\times \;\psi_{E,age}\left({Age\_group}_{E,i}\right)$$

$\varepsilon_{g}$ is the baseline rate of exposure from the community, which is estimated for each of the two RSV groups. $\psi_{E,age}$ is the coefficient modifying the rate of community exposure by age. For each RSV group, we have $f_{g}(t)$, a time-unit dependent curve that modifies the community rate of exposure over time, in this case the time period of interest is the duration of the study. We wanted this curve to represent the background epidemic dynamics in the local zone from which the data was collected; as such we proceeded to use the same household dataset to generate it.

The data are calibrated in days and are at the individual level, but to obtain the background community rate, we assumed that this background rate is scalable from the weekly household-level rate of primary incidence, denoted $\lambda_{HH}\left(t_{w}\right)$. The household level rate of primary incidence is the rate at which a household (rather than a single member of a household) acquires the first episode/outbreak in the ongoing RSV season. A household outbreak is a period within which at any given time, at least one household member is shedding RSV. If we treat $\lambda_{HH}\left(t_{w}\right)$ as the hazard rate in a probability distribution, we can estimate it using the following model:

$$I_{c}\left(t_{w}\right)=N_{HH}\left(1-\;{exp}^{-\int_{0}^{t_{w}}\lambda_{HH}\left(s\right)}\right)$$

$$\;I\left(t_{w}\right)=max\left(0,\;[I_{c}\left(t_{w}\right)-I_{c}\left(t_{w}-1\right)]\right)$$

Where

*N_HH_* = Total number of households in the study

*I(*$t_{w}$*)* = Average weekly household-level incidence of primary infection

*I_C_(*$t_{w}$*)* = Weekly cumulative household-level incidence of primary infection

We further assumed that $\lambda_{HH}\left(t_{w}\right)=a_{1}{exp}^{-{\left(\frac{t_{w}-b_{1}}{c_{1}}\right)}^{2}}$, giving it a bell-shape, and estimated {a_1_, b_1_, c_1_} using maximum likelihood assuming Poisson distributed data.

Once $\lambda_{HH}\left(t_{w}\right)$ was estimated for each RSV group, it was scaled such that it ranges between 0 and 1 using the formula $X_{i}^{Scaled}=\frac{X_{i}-min\left(\left\{X\right\}\right)}{\max\left(\left\{X\right\}\right)-min\left(\left\{X\right\}\right)}$. As such,

$$f_{g}\left(t_{w}\right)=\frac{\lambda_{HH}\left(t_{w}\right)-min\left(\left\{\lambda_{HH}\left(1\right),\lambda_{HH}\left(2\right)\ldots \lambda_{HH}\left(t_{w}\right)\right\}\right)}{\max\left(\left\{\lambda_{HH}\left(1\right),\lambda_{HH}\left(2\right)\ldots \lambda_{HH}\left(t_{w}\right)\right\}\right)-min\left(\left\{\lambda_{HH}\left(1\right),\lambda_{HH}\left(2\right)\ldots \lambda_{HH}\left(t_{w}\right)\right\}\right)}$$

**Fig. 1:**
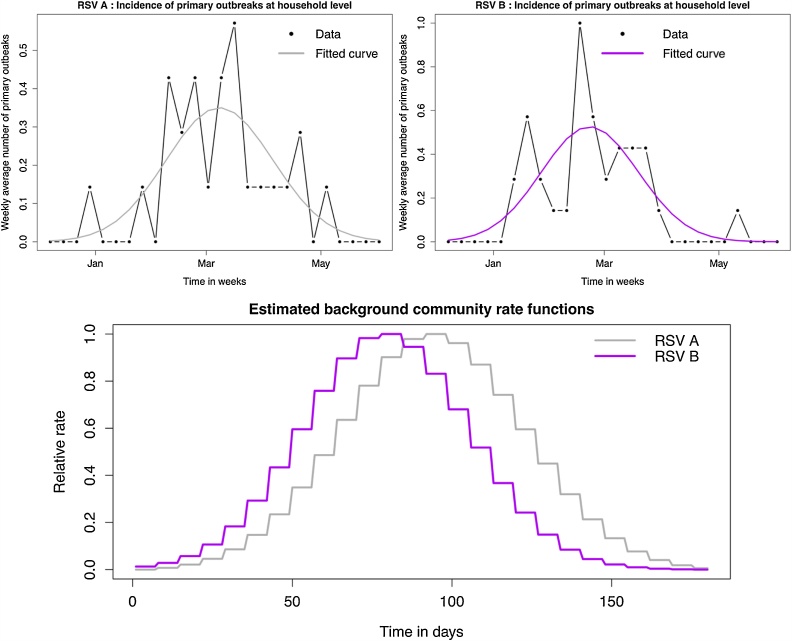
Establishing the background community rate function. The figures in the top row show a comparison of data and model fit of the weekly household-level rate of primary incidence that was used to derive the background community rate function. Top left: RSV A data and model fit; Top right: RSV B data and model fit; Bottom: Comparing the estimated background community rate function for RSV A and RSV B.

To turn $f_{g}\left(t_{w}\right)$ into a daily scale, the value for a given week were assumed to be the values for every day of that week. The resultant background community curves for RSV A and B are shown in Fig. 1.

Finally, we assume that susceptibility can be modified according to an individual’s infection history within the same epidemic, and their age. These two components are combined into an equation representing relative susceptibility to infection as shown below

$$S_{i,g}\left(t\right)=\exp\left(\phi_{Y,hist}\left({Infection\_History}_{i}\left(t\right)\right)+\;\phi_{X,age}\left({Age\_group}_{S,i}\right)\right)$$

$\phi_{X,age}$ is the coefficient modifying susceptibility by age. We categorized infection history into four groups: no previous infection, recovered from an RSV A infection, recovered from an RSV B infection, recovered from both RSV A and B. $\;\phi_{Y,hist}$ is the coefficient modifying susceptibility to a particular RSV group depending on infection history in the following three ways: by ${exp}^{\phi_{Y,hom}}$ if an individual has previously experienced and recovered from infection by the same group (homologous infection), ${exp}^{\phi_{Y,het}}$ if the individual has previously experienced and recovered from infection by a different group (heterologous infection) and by $\;{exp}^{{(\phi }_{Y,hom}+\phi_{Y,het})}$ if an individual has previously experienced and recovered from both RSV A and RSV B infection. This mechanism of interaction between RSV A and B is similar to that applied in a compartmental model used to analyze data from the UK and Finland (White et al., 2005).

In combination, all the above assumptions result in the rate of exposure equation shown below

1) $\lambda_{i,h,g}\left(t\right)$ = Rate of exposure of individual *i* in household *h* with RSV group *g* at time t.

$$\lambda_{i,h,g}\left(t\right)=S_{i,g}(t)\left[M_{i,h}(t)\sum_{j\neq i}{HH\_Risk}_{h,g,j\to i}\left(t\right)+\;{Comm\_Risk}_{i,g}(t)\right]$$

The assumption of how age and infection history modify the rate of exposure is similar to the assumptions made in a proportional hazards model.

Additional details on the data variables and parameters are given in Table 1.

**Table 1:** Model Notation.

Symbol	Name	Type	Description
*i*		Index	Index of individual
*h*		Index	Index of household
*g*		Index	Index of RSV group type, either A or B
*t*		Index	Index of time in days
$I_{j,h,g}\left(t\right)$	*Infectivity*	Data^a^	Categorical data variable for infectious individuals indicating level of infectivity categorized by viral load and symptom status at time *t*. The categories are: low viral load and asymptomatic (reference group), high viral load and asymptomatic, low viral load and symptomatic and, high viral load and symptomatic. High viral load is defined as >6 log_10_ viral copy number.
$Y_{i}\left(t\right)$	*Infection_history*	Data	Variable indicating if an individual has experienced and recovered from an infection by a particular RSV group in the current epidemic at time *t*.
$X_{i}$	*Age_group_S_*	Data	Categorical data variable indicating the susceptibility age group of an individual. The age groups are <1 year (reference group), 1-4 years, 5-14 years and ≥15 years.
$M_{i,h}\left(t\right)$		Data	Binary data variable indicating if an individual is present in the household at time *t*. Absence from the household means that an individual was not present at the point of sample collection and thus in the model they can only get infection from a community source and not from an infectious housemate (not sampled and not at household risk). Individuals who were present but not sampled are exposed to both household and community source transmission in the models (not sampled but at household risk).
$H_{i}$	Household_size	Data^a^	Binary data variable indicating whether the individual lives in a large or small household. A small household (reference group) has <8 individuals.
$E_{i}$	Age_group_E_	Data	Categorical data variable indicating the community exposure age group of an individual. The age groups are <1 year (reference group), 1-4 years and ≥5 years.
$\phi_{X,age}$	*Sus.age.2* *Sus.age.3* *Sus.age.4*	Parameter	Coefficients modifying susceptibility to RSV depending on age, applied to the age group covariate *X_i_*. *Sus.age.2* estimates the effect being in age group 1-4 years, *Sus.age.3* the effect of group 5-15 and *Sus.age.4* of group ≥15 relative to group <1 year.
$\phi_{Y,hist}$	*Prev.hom* *Prev.het*	Parameter	Coefficients modifying susceptibility to infection by a particular RSV group depending on infection history. *Prev.hom* estimates the effect of a previous homologous group infection, while *Prev.het* estimates the effect of a previous heterologous group infection. Applied to the categorical covariate *Y_i_(t)*.
$\psi_{H}$	*HH.size*	Parameter	Coefficient modifying the amount of within household exposure by household size. *HH.size* estimates the effect of being in a large household relative to a small one. Applied to covariate *H_i_*.
$\eta_{g}$	*HH.rsv.a* *HH.rsv.b*	Parameter	Baseline rate of within household exposure by RSV group
$\psi_{I,inf}$	*High.Asym* *Low.Sym* *High.Sym*	Parameter	Coefficients modifying infectiousness by viral load and symptom status. Relative to shedding low viral load and being asymptomatic, *High.Asym* estimates the effect of shedding high viral load and being asymptomatic, *Low.Sym* the effect of shedding low viral load and being symptomatic and *High.Sym* the effect of shedding high viral load and being symptomatic. Applied to the infectivity covariate $I_{j,h,g}\left(t\right)$.
$\psi_{E,age}$	*Exp.age.2* *Exp.age.3*	Parameter	Coefficients modifying the rate of community exposure by age group. *Exp.age.2* estimates the effect being in age group 1-4 years and *Exp.age.3* the effect of group ≥5, relative to the <1-year age group. Applied to the age group covariate *E_i_*
$\varepsilon_{g}$	*Comm.rsv.a* *Comm.rsv.b*	Parameter	Community transmission coefficient by RSV group
$f_{g}(t)$		Estimated	RSV group specific, time-dependent curve modifying the rate of community exposure.
$U_{i,h,g}$		Data	Set of group specific onset days for an individual *i* in household *h* used in calculating the likelihood of an individual’s data.

a The choice of cut-off for high viral load and large households was based on initial runs of the inference algorithm that explored different cut-offs for each. The choice of 6 log_10_ copy number for high viral load and 8 persons for large households led to the best convergence.

Following on from the rate of exposure equation are two additional nested equations that make up the model.

2) $\alpha_{i,h,g}(t)$ = Probability of infection following exposure per day i.e. individual enters the latent phase

$$\alpha_{i,h,g}(t)=\left(1-{exp}^{-\lambda_{i,h,g}(t)}\right)$$

3) $p_{i,h,g}(t)$ = Probability of starting to shed i.e. individual enters the infectious phase

$$p_{i,h,g}(t)=\sum_{l=0}^{L}\theta_{l}\alpha_{i,h,g}(t-l)$$

Where L is the maximum latent period and $\theta_{l}$ is the probability that the latent period is exactly l days. For l = {0,1,2,3,4,5} days, we have the following probabilities [0,0,4,4,3,1]/12= [0, 0,0.33,0.33,0.25,0.083] (Lee et al., 2004). The same latency distribution is used for RSV A and B.

The likelihood of an individual’s data, given the above model thus becomes:

$$L_{i}=\;\prod_{g}\left[\prod_{u\in U_{i,h,g}}p_{i,h,g}(u)\prod_{u\notin U_{i,h,g}}\left(1-p_{i,h,g}(u)\right)\right]$$

The model as presented can be reduced to fit for a single RSV group or for RSV as a single pathogen with no distinction between RSV A and B. Attempts to model household size as a continuous variable were unsuccessful possibly due to our small sample size and hence we modeled transmission within the household as a density dependent process but identified households as either large or small and found that the cut-off between categories of 8 provided the best fit.

### 2.3 Parameter inference

We used Bayesian inference to obtain estimates of the parameters. Adaptive Metropolis Markov Chain Monte Carlo was used as implemented in the R software package *fitR* (Camacho and Funk, 2017), function *mcmcMH*. The *mcmcMH* function can adapt the size of the proposal distribution, such that the acceptance rate is close to 23.4%, and the shape using the Adaptive metropolis algorithm as in (Roberts and Rosenthal, 2009); the difference in size and shape adaptation being in the scaling factor used. In brief, the method builds a Markov chain which allows us to sample from the posterior distribution *P(φ|D)* of the parameters given the data, where *φ*={$\phi_{X,age}$, $\;\phi_{Y,hist}$, $\;\psi_{H}$, $\;\eta_{g}$, $\;\psi_{I,inf}$, $\;\psi_{E,age}$, $\;\varepsilon_{g}$}. Flat bounded priors were used for all the parameters. We initiated 3 chains and set the algorithm to start adapting the size of the proposal distribution after 1000 iterations and the shape after 500 accepted iterations.

Burn-in was assessed visually after which the results of the three concurrent chains were combined to infer the posterior distribution. To obtain fairly accurate values for the 95% credible intervals, we ran the MCMC algorithm until the effective sample size (ESS) was ≥ 4000 (Raftery and Lewis, 1992). The three chains were run for 250,000 iterations each and burn-in for each chain was 80,000, 90,000 and 80,000. After burn-in the reminders of the three chains were combined into a single chain with and overall acceptance rate of 16.8%. The parameters were estimated on the log scale. All the computation was done using R software package (RStudio version 1.1.383 running R version 3.4.0 (R Core Team, 2017)). The code is freely available under the GNU Lesser General Public License v3.0 and can be found at https://github.com/Ikadzo/HH_Transmission_Model.

## 3 Ethics statement

For the data collection, informed written consent was obtained from all the study participants or their parents/guardian. The KEMRI-Scientific and Ethical Review Committee in Kenya provided ethical approval. The analysis presented here falls under the expected results from the original data collection study, however, additional ethical approval was obtained from the Observational / Interventions Research Ethics Committee at the London School of Hygiene and Tropical Medicine.

## 4 Results

**Fig. 2:**
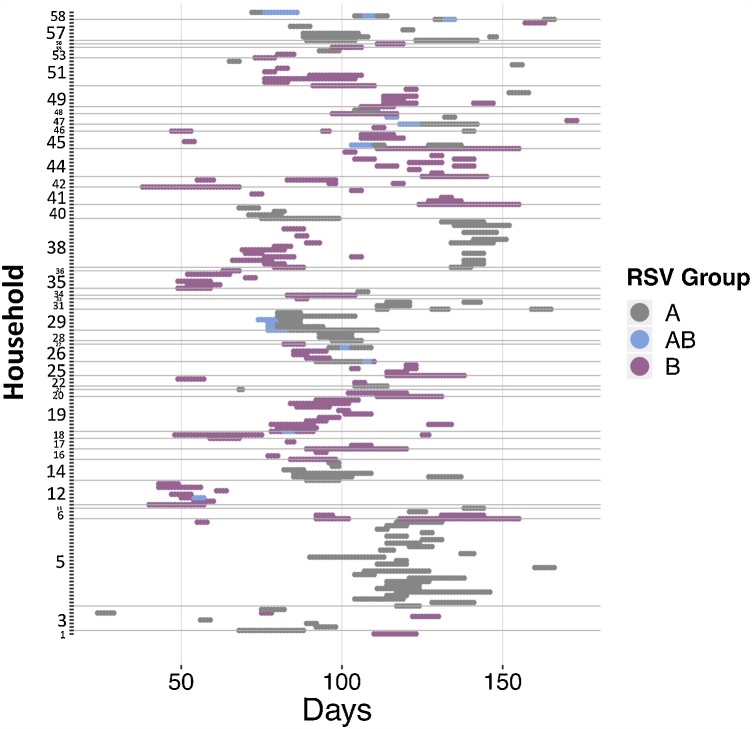
Shedding patterns for each of the 179 individuals who experienced at least one RSV shedding episode. The y-axis shows the household, time is on the x-axis with zero indicating the day before the first sample was collected. The grey dots show RSV A shedding, dark pink show RSV B and blue shows days of co-shedding. The horizontal grey lines separate the data by household. The study initially recruited 60 households but 13 were lost to follow-up, hence the numbering of the households goes beyond 47. (For interpretation of the references to colour in this figure legend, the reader is referred to the web version of this article).

Table 2 gives a summary of the shedding episodes in the data. This particular outbreak had more RSV B cases than RSV A, with a significant portion of cases being symptomatic both for RSV A and B. Eighty five percent of the households that were successfully followed up had an introduction of an RSV case. In addition to the information in Table 2; 28 (13.5%) of the total 208 episodes were censored during imputation; of the A and B episodes, 14 (6.7%) were simultaneous RSV A and B shedding episodes, 7 (3.3%) of which had a simultaneous onset; of the 179 individuals who got infected 31 (17.3%) were <1 year old, 41 (22.9%) were 1–4 years, 66 (36.9%) were 5–14 years and 41 (22.9%) ≥15 years old. Of the symptomatic infected individuals, 28 (25.7%) were <1 year old, 35 (32.1%) were 1–4 years, 36 (33%) were 5–14 years and 10 (9.2%) ≥15 years old. A detailed analysis of these shedding patterns has been published elsewhere (Wathuo et al., 2016). Fig. 2 shows the shedding pattern for all 179 people who had a shedding episode. Figure A.3 and Figure A.4 in the Supplementary appendix shows the shedding and ARI patterns for RSV A and B respectively.

**Table 2:** Summary of shedding episodes.

	RSV A	RSV B	All RSV
Number of episodes	97	125	208
Number of symptomatic episodes	59	69	119
Number of people infected	88	113	179
Number of people with symptomatic episodes	54	67	109
Number of people with repeat infections	8	12	27
Number of households infected (percentage of total)	25 (53.2%)	34 (72.3%)	40 (85.1%)
Total percentage of household occupants that were infected (total number of occupants)^a^	30.0% (293)	28.5% (396)	40.5% (442)

a The total number of infected individuals out of the total number of individuals that occupy the infected households.

### 4.1 Transmission model parameter inference

The trace plots used to assess convergence of the three chains are shown in Figure A.5 in the Supplementary appendix. The resulting parameters estimates are given in Table 3 and Figure A.6.

**Table 3:** Results of fitting the transmission model. Median and 95% credible intervals (CrI) are given for the 15 parameters of interest. The posterior distribution for each parameter was obtained by running 3 MCMC chains for 250,000 iterations each. The burn-in for the three chains was 80,000, 90,000 and 80,000 respectively. The reminders of the three chains were combined into a single chain with and overall acceptance rate of 16.8%.

Parameter name	Median	95% credible interval (CrI)
*Prev.hom*	0.530	0.316 - 0.833
*Prev.het*	0.607	0.306 - 1.08
*Sus.age.2*	0.924	0.483 - 1.87
*Sus.age.3*	0.267	0.142 - 0.537
*Sus.age.4*	0.155	0.0825 - 0.316
*HH.rsv.a*	0.0188	0.00734 - 0.0401
*HH.rsv.b*	0.015	0.00578 - 0.033
*HH.size*	0.424	0.265 - 0.702
*High.Asym*	0.0704	0.0000692 - 3.15
*Low.Sym*	2.48	1.22 - 5.57
*High.Sym*	6.7	2.56 – 16.0
*Comm.rsv.a*	0.00338	0.00203 - 0.00530
*Comm.rsv.b*	0.00615	0.00388 - 0.00926
*Exp.age.2*	0.563	0.206 - 1.45
*Exp.age.3*	1.87	0.788 - 4.26

In short, susceptibility to infection was reduced by previous infection whether these infections were homologous (*Prev.hom* = 0.53 (0.32 - 0.83)) or heterologous (*Prev.het* = 0.61 (0.3–1.1)). Increasing age also reduces susceptibility with ages 1–4 years old having an estimated 8% reduction (*Sus.age.2* = 0.92 (0.48–1.9)), ages 5–15 years a 73% reduction (*Sus.age.3* = 0.27 (0.14 - 0.53)) and ages ≥15 years an 84% reduction (*Sus.age.4* = 0.16 (0.08 - 0.32)). The within household transmission coefficients (*HH.rsv.a* = 0.019 (0.0073 – 0.04) and *HH.rsv.b* = 0.015 (0.0058 – 0.033)) are estimated higher than the community transmission coefficients (*Comm.rsv.a* = 0.0034 (0.002 – 0.0053) and *Comm.rsv.b* = 0.0062 (0.0039 – 0.0093)). The coefficient modifying within household exposure by size (*HH.size* = 0.42 (0.27 – 0.7)) suggests that larger households have less risk of pair-wise within household transmission (${HH.Risk}_{h,g,j\to i}\left(t\right)$) than smaller households. However the total risk of household transmission ($\sum_{j\neq i}{HH.Risk}_{h,g,j\to i}\left(t\right)$) is in general higher for larger households as they can have more infectious individuals at a time point, this is illustrated in Figure A.7.

Although there is suggestion that pre-school individuals are the least likely to acquire infection from the community, and school-age individuals and older are the most likely to acquire community infection, the evidence is very weak: the relative estimate for age groups 1–4 years is *Exp.age.2* = 0.56 (0.21–1.5) while for age group ≥5 years is *Exp.age.3* = 1.9 (0.78–4.2). Symptomatic individuals are more infectious than asymptomatic individuals, more so those with high viral load, the relative estimate for high viral load symptomatic shedders is given as *High.Sym* = 6.7 (2.6–16). However there are not enough instances where individuals have high viral load and are asymptomatic to quantify the relative infectiousness of this specific combination, the relative estimate for high viral load asymptomatic shedders, *High.Asym*, has a very wide 95% CrI. Given 71,132 person days of observation (493 individuals * 180 days of data, minus days individuals were away), 1021 had RSV A shedding, of which 49 were asymptomatic high viral load shedding days, and 1227 had RSV B shedding with 49 days of asymptomatic high viral load shedding. Given the inability to distinguish between the infectiousness of high versus low viral load asymptomatic shedders, we will not make this distinction in subsequent results and instead just refer to asymptomatic shedders in general.

**Fig. 3:**
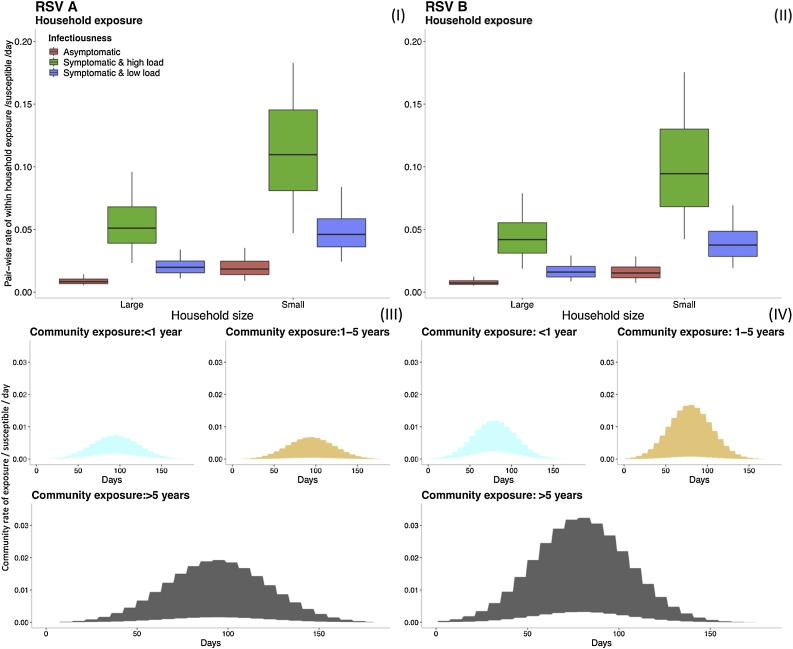
Comparing the range of within household exposure rate $\left({HH\_Risk}_{h,g,j\to i}\left(t\right)\right)$, (I) and (II), and community exposure rate $\left({Comm\_Risk}_{i,g}\left(t\right)\right)$, (III) and (IV), for a single susceptible individual given different heterogeneities in exposure and infectiousness. Top row: The box plots show the 0.025, 0.25, 0.5, 0.75 and 0.975 percentiles for the rate of exposure per person per day between a single susceptible and a single infectious housemate for RSV A (I) and RSV B (II). The distributions of rate are categorized by household size and the infectiousness based on viral load and symptom status (see text). Note: outliers have been removed from the box plots for better visualization. Bottom row: The shaded graphs show the range of values over time for the rate of exposure from the community to a single susceptible individual for RSV A (III) and RSV B (IV). The graphs are color-coded by the age group of the susceptible individual. The ranges for each age group are determined by the 95% CrI of the parameters that go into the calculations, hence the shaded regions show 95% CrI of the community exposure rate.

For a better understanding of the within household and community transmission coefficient parameters, we calculated the different rates of exposure and plotted them as shown in Fig. 3.

Given two competing sources of infection, an infectious housemate and a source outside of the household, a susceptible individual is more likely to get infected within the household rather than from the community. There is a suggestion that RSV A has a higher transmission potential at the household level relative to RSV B, while the situation is reversed at the community level. However, there is considerable overlap between the distributions of within household transmission coefficient for RSV A and that for RSV B as seen in Figure A.6, which shows the distribution of the parameters on the log scale, which is mirrored in the rate of household exposure shown in Fig. 3.

We observed some correlations in the estimated parameters. In particular there were strong positive correlations within the relative susceptibility by age parameters. The within household transmission coefficient for RSV A was strongly positively correlated with the within household transmission coefficient for RSV B. The age effects of susceptibility were strongly negatively correlated with the age effects on community exposure. Figure A. 8 in the Supplementary index shows all the pairwise correlation patterns.

Given the posterior densities for the parameters, we calculated the source with the highest likelihood for each infection. While respecting the correlation patterns observed in Figure A.8, we sampled 10 different parameter sets and for each, we calculated the proportion of cases whose most likely source was an infectious housemate. The changes made to the likelihood equation to allow for this calculation are described in the Supplementary appendix. For all the infection cases, 32–53% of them were attributed to transmission within the household. For RSV A, this range was 40–59%, while for RSV B it was 26–48%.

To check if any information is lost when we have less data, we refitted the data in three additional ways: RSV A alone, RSV B alone and RSV with no distinction between groups. The results are shown in Table A. 1 in the Supplementary index. In reducing the data used to infer parameters we notice that more posterior densities for the relative effect parameters now include 1 in their 95% credible interval, as can be expected. In general, the trends with age, household size and relative infectiousness, as seen in Figure A.6, are maintained. However, when RSV is treated as one entity, the protective effect of previous infection is reduced, symptomatic cases are more infectious and the estimate of the community transmission coefficient is increased. This suggests that misclassification of viruses disrupts the ability of the model to track transmission patterns, resulting in a greater propensity to account for infections as spontaneous.

### 4.2 Model validation and sensitivity analysis

**Fig. 4:**
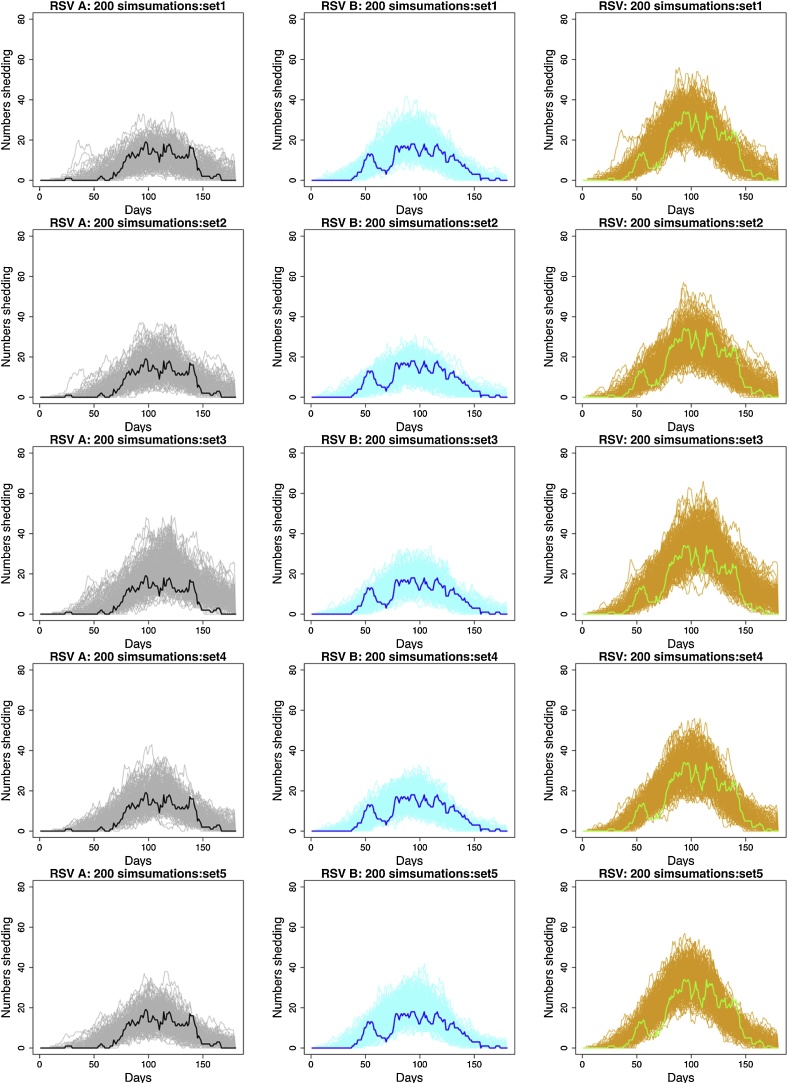
A comparison between the simulated data and real epidemics using simulations from 5 different parameter sets estimated from the full model (row 1 to 5). First column: RSV A simulated epidemics (grey lines) compared to real data (thick black line). Second column: RSV B simulated epidemics (light blue lines) compared to real data (thick blue line). Third column: RSV simulated epidemics (orange lines) compared to real data (thick green lines) (For interpretation of the references to colour in this figure legend, the reader is referred to the web version of this article).

To validate the model we checked to see that the range of simulated epidemics contained the real data; then we chose a single simulation with known parameters and re-estimated to see if the posterior distribution contained the known values. Details of this process can be found in the Supplementary appendix, but in general, we were satisfied that the model was working as expected. Fig. 4 shows multiple simulated epidemics for different parameter sets relative to the real data. From this we see that as with the real data, the simulations show the RSV B epidemic taking off earlier than the RSV A epidemic. There is a tendency for simulate epidemics to be larger than that observed in terms of total number of cases (Figure A.23).

We performed a sensitivity analysis to check the robustness of our results to the background community density function. We used 3 additional background functions and found that despite a change in summary values for the parameters, in general the trends were maintained. These results are shown in the Supplementary appendix. They show that the results are robust to the choice in the shape of background community density function.

Finally, we removed the largest household (which had a very large RSV A outbreak but only a single RSV B case) from the data to check if this would change the patterns of the within household transmission coefficients. The results, shown in the Supplementary appendix, were robust to these changes.

Following the validation of the model, we simulated epidemics altering the degree of infectiousness. Initially we reduced the infectiousness of symptomatic individuals to predict the effect of reducing RSV related ARI; then we assumed that asymptomatic individuals are not infectious in order to quantify the contribution of asymptomatic infections to transmission. The results show that reducing infectiousness of symptomatic individuals to the level of asymptomatic individuals lowers the distribution of total number infected. Assuming that asymptomatic individuals are not infectious also tends to decrease the total number infected (see Figure A. 23 in the Supplementary appendix). We also removed the asymptomatic shedding episodes from the data and re-estimated the parameters to check what the effect of only having sampled symptomatic individuals would be. We found that we lose precision in the estimates of the relative infectiousness parameters, previous infection is estimated as being more protective as is being ≥15 years old (Figure A. 24 and A.25).

## 5 Discussion

We developed an individual based approach to make Bayesian based inference on transmission parameters using MCMC. We set out to better understand RSV transmission within a household setting using cohort data collected with unprecedented detail during the course of a single RSV epidemic in a rural coastal community in Kenya.

Older individuals are less susceptible to detectable infection, presumably due to immunity acquired in previous epidemics. We found strong evidence of partial immunity to homologous re-infection within the same epidemic for the RSV groups. The effect of previous infections is captured in two different ways in our model. Age (*Sus.age* parameters) captures the combined effect of age and experience of epidemics prior to the one under study, while the estimates for the effect of previous observed infections (*Prev.hom* parameter), captures effect of infections in the current epidemic. It is therefore implicit that immunity to RSV is built up in the long term, from one epidemic to the next and in the short term from one infection to the next. The evidence for cross-immunity between RSV A and B was weaker, which presumably allowed the two virus groups to co-circulate in this epidemic. However, typically, RSV epidemics are dominated by one or other of group A or B and so the particular circumstances of this epidemic might not always hold. It remains to be explored how this individual level parameter estimate is translated into population dynamics.

We found some evidence that individuals aged ≥5 years were the most likely to get infection from a community source (less likely to get infected during a household outbreak). This means that given our assumption of latent periods between 2–5 days, which forms the temporal link between cases, individuals ≥5 years were the most often identified as index cases in a household outbreak relative to the younger age groups. We have not considered an age-dependent latent period, and estimating the latent period from these data is a future goal. The ≥5 years age group contains school going children and our result is in line with those of Munywoki et al (Munywoki et al., 2014), based on a different analysis of the same study, who found that school-going children were often initiating household outbreaks. Establishing transmission chains using genomic information could strengthen this result.

We have assumed that the community risk of infection changes smoothly over time and is homogeneous apart from an age effect. These assumptions are necessary as community infections are not completely observed. We are confident that these assumptions do not have significant influence on our estimates of within-household transmission (which is fully observed), but may result in an over-estimate of community exposure, which will be more heterogeneous than we have assumed. Consequently the simulated epidemics are larger in total numbers than that observed, Fig. 4, and our results of up to one half of infections arising from within the household are likely to be a minimum. Data on genetic relatedness between viral isolates will clarify the extent to which individuals are infected from the community during a household outbreak.

By separating RSV A and RSV B we find that RSV B has a higher rate of introduction into the household, and RSV A is more transmissible once in the household, an observation also made by (Agoti et al., 2017) from a phylogenetic analysis of RSV A sequences. This, together with the fact that RSV A had a larger proportion of cases attributed to within household transmission, suggests that there might be some niche separation, explaining how and why these two different groups are able to co-exist and remain separate. It should be noted however that the difference in the distribution of the within household transmission coefficient between the RSV groups is not large, there is a significant overlap of credible intervals. As such, whatever advantage RSV A might have over RSV B at the household level is small in terms of transmission, but might be larger in terms of interaction with other respiratory viruses, and small differences in individual based parameters might translate into large population effects. In the present epidemic, the RSV B epidemic takes off earlier than the RSV A epidemic despite the first case being RSV A (Fig. 2). In addition to which, we see that despite RSV B infecting more households than RSV A, RSV A infects a larger proportion of household members (Table 2). An examination of the comparative dynamics of RSV A and B within epidemics might be a good way to understand how they interact.

With the definition of a household as a group of individuals living in the same compound and eating food from the same kitchen, we found that the pairwise rate of within household transmission is higher in small households than large ones. However, the total household incidence rate is in general higher for larger households as they have the potential to have larger numbers of infectious individuals at a given time point. The relationship between household size and pair-wise rate of transmission has been observed before for Influenza, (Cauchemez et al., 2009, 2004; House et al., 2012; Lau et al., 2015), however going a step further we show that if households are structured such that they can hold over 20 individuals (possibly several members of an extended family as is the case in the present study) then larger households will tend to contribute more to transmission than smaller households.

We looked at a combination of presence of symptoms and viral load to infer infectiousness. We found that being symptomatic is of key importance. In general, symptomatic individuals were more infectious, particularly if shedding large amounts of virus. Though this result is not surprising it has an important implication on vaccine effectiveness. If an RSV vaccine works by reducing or preventing disease in the form of an ARI, this will in turn have an impact on transmission potential and we should expect to see reduced morbidity and infection. To check what that potential impact of such a vaccine would be, we simulated epidemics where the infectiousness of symptomatic individuals was equal to that of asymptomatic individuals and we found a significant shift in the overall distribution of simulated case towards smaller total numbers infected. The shift was more for ages between 1 and 15 years, given that this group also had the larger fraction of symptomatic cases, the observation from simulations with reduced infectiousness suggests largely assortative mixing within this group, which in turn means largely assortative transmission. The number of cases in the <1 year age group is not greatly altered by reducing the infectiousness of symptomatic individuals, implying that there are several sources of infection to the infant and reducing or removing only one has little impact (Figure A. 23).

We reduced the model complexity to look at RSV as a single pathogen without distinguishing between groups. This resulted in skewing the parameter estimates away from within household transmission and towards spontaneous infection from external sources, as a result of introductions due to RSV A and RSV B being treated as multiple introduction of the same pathogen thus compounding the effect of community transmission. This, in addition to the reduced protective effect of previous infection due to misclassification of re-infections, led to the within household transmission parameter being underestimated in order for the model to account for the observed number of infections. In addition, temporally linking RSV A and B cases as a result of misclassification also led to the effect of symptoms on transmission being overestimated. This suggests that the estimates obtained in the present analysis are likely to change if we further classified the cases into RSV subgroups. This goes to illustrate the importance of making distinctions between pathogens in order to obtain accurate estimates of transmission parameters. At any given moment multiple pathogens are co-circulating in a host population, this household study alone had multiple viruses spreading in large numbers during the time of data collection (Munywoki et al., 2018). How these pathogens interact could have dramatic implications for parameter estimates, and ultimately on how control strategies are implemented. We have seen the effect of the pneumococcal vaccine on the non-vaccine serotypes and how it might mitigate vaccine effectiveness (Kwambana-Adams et al., 2017) and a study on influenza has shown evidence of its controlling effect on other pathogens (Zheng et al., 2017). There is an increasing call from such observations to understand how multiple pathogens interact at the host population level.

Our study is not without limitations. The households in the study were selected based on the presence of an infant born after the previous RSV epidemic and older siblings to the infant in order to determine who infects the infant. As such the sample is not random and this might introduce bias in the parameter estimates, the extent of which we are uncertain. Relative to other studies, our sample size in terms of number of households is small. However, the intensive sampling regardless of symptoms means we had less biased observation of infections relative to index-case ascertained household studies that rely on symptom reporting by household contacts. In our study we had 47.2% of RSV A and 40.2% of RSV B positive samples that were symptomatic, 60.8% of RSV A and 55.2% of RSV B episodes were symptomatic. Estimation of parameters only using data from symptomatic episodes shows similar parameter estimates, although with loss of precision, especially in terms of differential infectiousness (Figure A. 24). In addition, sampling was done every 3 or 4 days, which means that short duration infections might have been missed, and we do not have serological data to complement the PCR results.

The present analysis could be extended in several ways. We used interpolated shedding durations; it would be an added advantage to use the data to estimate a distribution of shedding durations that could potentially be more generalizable. The inclusion of other sources of information into the analysis could improve parameter inference, as was the case with Li et al and the inclusion of genetic data (Li et al., 2017). The inference made on within-household transmission compared to community transmission is based on the latency distribution that links onset of cases. This is a temporal linking of cases that is not always correct. A combination of temporal and genetic distance would allow better inference on linked cases and consequently the competition between within-household and community source transmission. Finally the RSV A and B model could be used to look at other pathogen interactions and perhaps incorporate more than two pathogens.

In conclusion, our analysis presents the first transmission modelling of cohort data for RSV and we find that it is important to factor in household size and social structuring – such as the tendency for households to contain several members of the extended family – when modelling transmission. It is also important to model competing risks of infection from within the household and the community. There are questions on the mechanisms that allow co-existence of RSV groups temporally and geographically. The weak cross immunity between RSV groups demonstrated by our analysis and the possibility of different transmission niches could form part of the explanation for the co-existence.

## Declaration of interest

None.

## Funding

This work was supported through the DELTAS Africa Initiative [DEL-15-003]. The DELTAS Africa Initiative is an independent funding scheme of the African Academy of Sciences (AAS)’s Alliance for Accelerating Excellence in Science in Africa (AESA) and supported by the New Partnership for Africa’s Development Planning and Coordinating Agency (NEPAD Agency) with funding from the Wellcome Trust [107769/Z/10/Z, 102975 and 090853] and the UK government. The views expressed in this publication are those of the authors and not necessarily those of AAS, NEPAD Agency, Wellcome Trust or the UK government.
