# Supplementary material for: Model-based estimates of transmission of respiratory syncytial virus within households
Source: Epidemics. 2019 Jun;27:1–11. doi: 10.1016/j.epidem.2018.12.001 (PMC6543068; doi:10.1016/j.epidem.2018.12.001)
Supplement: Supplementary file 1 [file mmc1.docx]

**Supplementary appendix**

**Model-based estimates of transmission of respiratory syncytial virus within households.**

Ivy K. Kombe^a, b*^, Patrick K. Munywoki^a^, Marc Baguelin^c^, D. James Nokes^a, d^, Graham F. Medley^b^

1. KEMRI-Wellcome Trust Research Programme, KEMRI Center for Geographical Medical Research-Coast. P.O. Box 230-80108, Kilifi, Kenya.
2. Centre for Mathematical Modelling of Infectious Disease and Department of Global Health and Development, London School of Hygiene and Tropical Medicine. London, WC1H 9SH, UK
3. Centre for Mathematical Modelling of Infectious Disease and Department of Infectious Disease Epidemiology, London School of Hygiene and Tropical Medicine. London, WC1H 9SH, UK
4. School of Life Sciences and Zeeman Institute for Systems Biology & Infectious Disease Epidemiology Research, University of Warwick. Coventry, CV4 7AL, UK

* Corresponding author

Email: [ikombe@kemri-wellcome.org](mailto:ikombe@kemri-wellcome.org) (Ivy K. Kombe)

Table of Contents

Imputing shedding durations, symptomatic episodes and viral loads 3

Extra results 8

Modification of the likelihood to establish the most likely infection source for every case. 14

Model validation 15

Sensitivity analysis 23

Checking the contribution of symptomatic and asymptomatic individuals 28

Fitting household size as an ordinal variable 32

References 34

# Imputing shedding durations, symptomatic episodes and viral loads

An RSV A/B shedding episode is defined as a period within which an individual provided PCR positive samples for RSV A/B that were no more than 14 days apart. Sampling of the study population was done in intervals, as such, complete shedding episodes had to be imputed using the mid-point method described. Shedding was assumed to start mid-way between the last negative sample and the first positive sample, and it ended midway between the last positive sample and the first negative sample of an episode. This is illustrated below:

*L=t_4_*

*J=t_3_*

*I=t_2_*

*K=t_1_*

*Time*

*Green circles are positive samples in a single episode, empty circle are negative. t_1_, t_2_, t_3_ and t_4_ are dates of sample collection.*

For (*t_4_*-*t_3_*) and (*t_2_*-*t_1_*) ≤7 days

$Duration=\left[ t_{3}+\left( \frac{t_{4}-t_{3}}{2} \right) \right]-\left[ t_{2}-\left( \frac{t_{2}-t_{1}}{2} \right) \right]$

For (*t_4_*-*t_3_*)>7

$Duration=\left[ t_{3}+\left( \frac{x}{2} \right) \right]-\left[ t_{2}-\left( \frac{t_{2}-t_{1}}{2} \right) \right]$ : Right censoring

For (*t_2_*-*t_1_*) >7

$Duration=\left[ t_{3}+\left( \frac{t_{4}-t_{3}}{2} \right) \right]-\left[ t_{2}+\left( \frac{x}{2} \right) \right]$ : Left censoring

Where x=mean of sampling intervals for samples in an episode, which was found to be 3.45 days.

Any negative samples (Ct >35 or Ct=0) in between a shedding episode were ignored, i.e. were not treated like true end of shedding. Figure A. 1 shows the distribution of imputed shedding durations for RSV A and RSV B episodes.


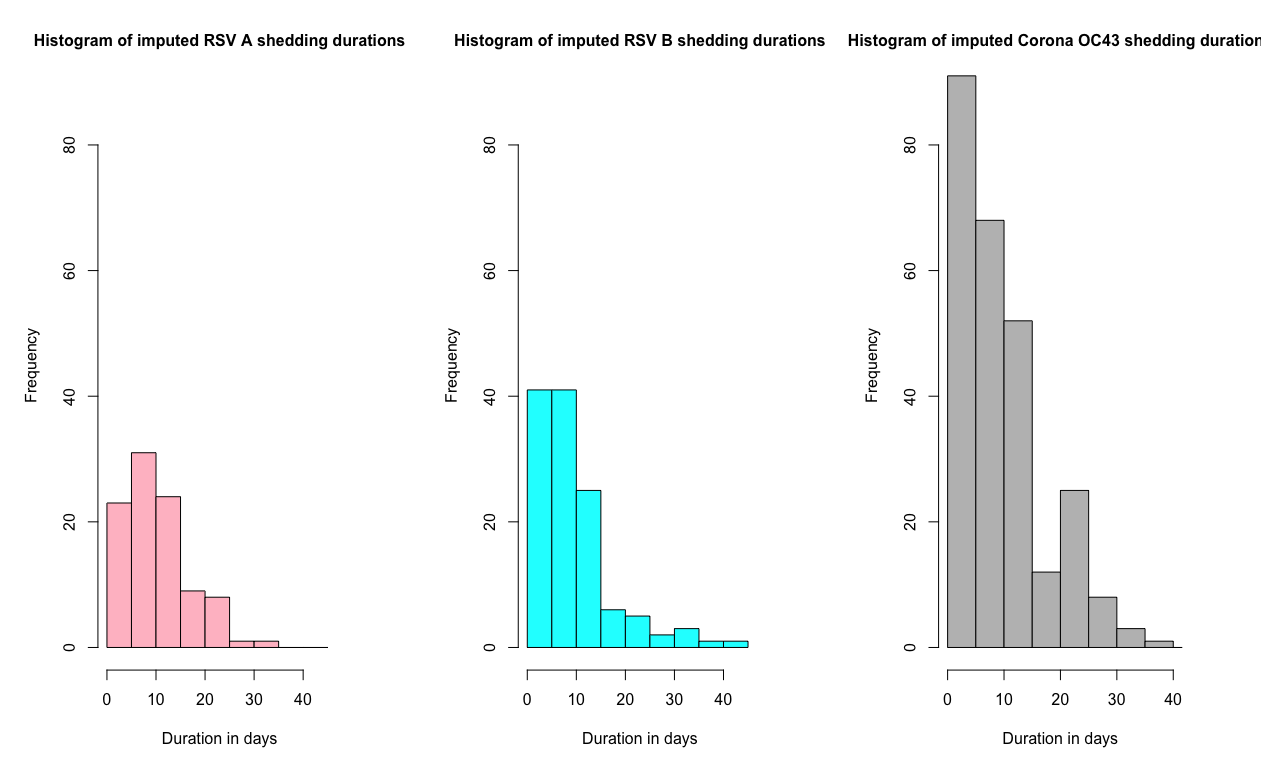


Figure A. 1: Distributions of imputed shedding durations for RSV A (left) and RSV (right)

In order to include information of the amount of virus shed by an infected person into the transmission model, the Ct value need to be converted to log_10_ RNA copy number which is a more direct measure of viral load. The formula used to convert Ct values to their log_10_ RNA equivalent was y= -3.308x + 42.9, where y=Ct values and x=log_10_ RNA copy number(Nolan et al., 2006; Wathuo et al., 2017).

Following conversion of the PCR Ct values to viral load, we proceeded to interpolate the viral loads for days in an episode that did not have data. Linear interpolation was used for all the shedding episodes. It was assumed that the starting and ending sample, if data was missing, had a viral load of 2.388 log_10_ RNA (baseline positive Ct value converted to viral load). For two samples of viral load *V_a_* and *V_b_* at times *t_a_* and *t_b_*, *t_b_* > *t_a_*, the gap in between is filled out as follows:

For *t_b_* – *t_a_* =n, viral load *V_j_* at time point *t_j_* for j=1…(n-1) is given by

$V_{j}=V_{a}+\frac{j \left( V_{b}-V_{a} \right)}{n}$

Viral loads lower than 2.388 log_10_ RNA in between an episode were not included in the interpolation. Figure A. 2 shows histograms of interpolated viral loads for RSV A and RSV B.


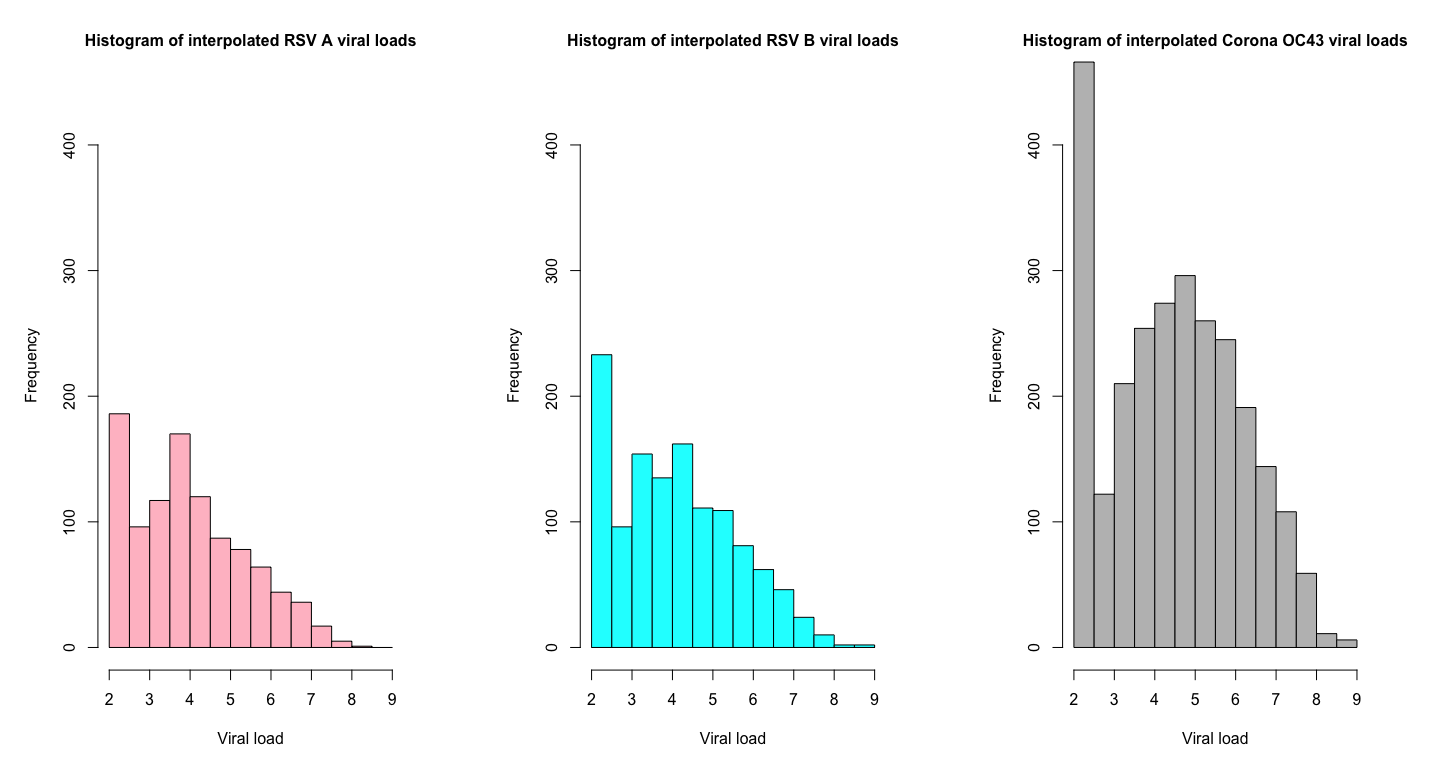


Figure A. 2: Histograms of interpolated viral loads for RSV A (left) and RSV B (right).

We define symptomatic as having an acute respiratory illness (ARI), which is defined as having at least one of three traits: cough or nasal discharge/ blockage or difficulty breathing. Within virus shedding episodes, we imputed complete ARI episodes from intervals of recorded ARI. A virus shedding episode that had no day where an ARI was reported was assumed to be asymptomatic. For a virus shedding episode with at least one day of recoded ARI, the duration of symptoms was imputed using the midpoint method described for shedding episodes. This is illustrated below:

*Green open circles are reported ARI symptoms (ARI positive) within the shedding episode and black open circles are confirmed absence of ARI (ARI negative). τ_1_, τ_2,_ τ_3_ and τ_4_ are days within the shedding episode where information on symptoms was collected.*

In this case, the mean sampling interval for ARI ‘samples’ within an episode was 3.78 days. This was obtained from all ARI episodes not just the ones within shedding episodes. Figure A. 3 and Figure A. 4 show the shedding patterns by RSV group and ARI status.


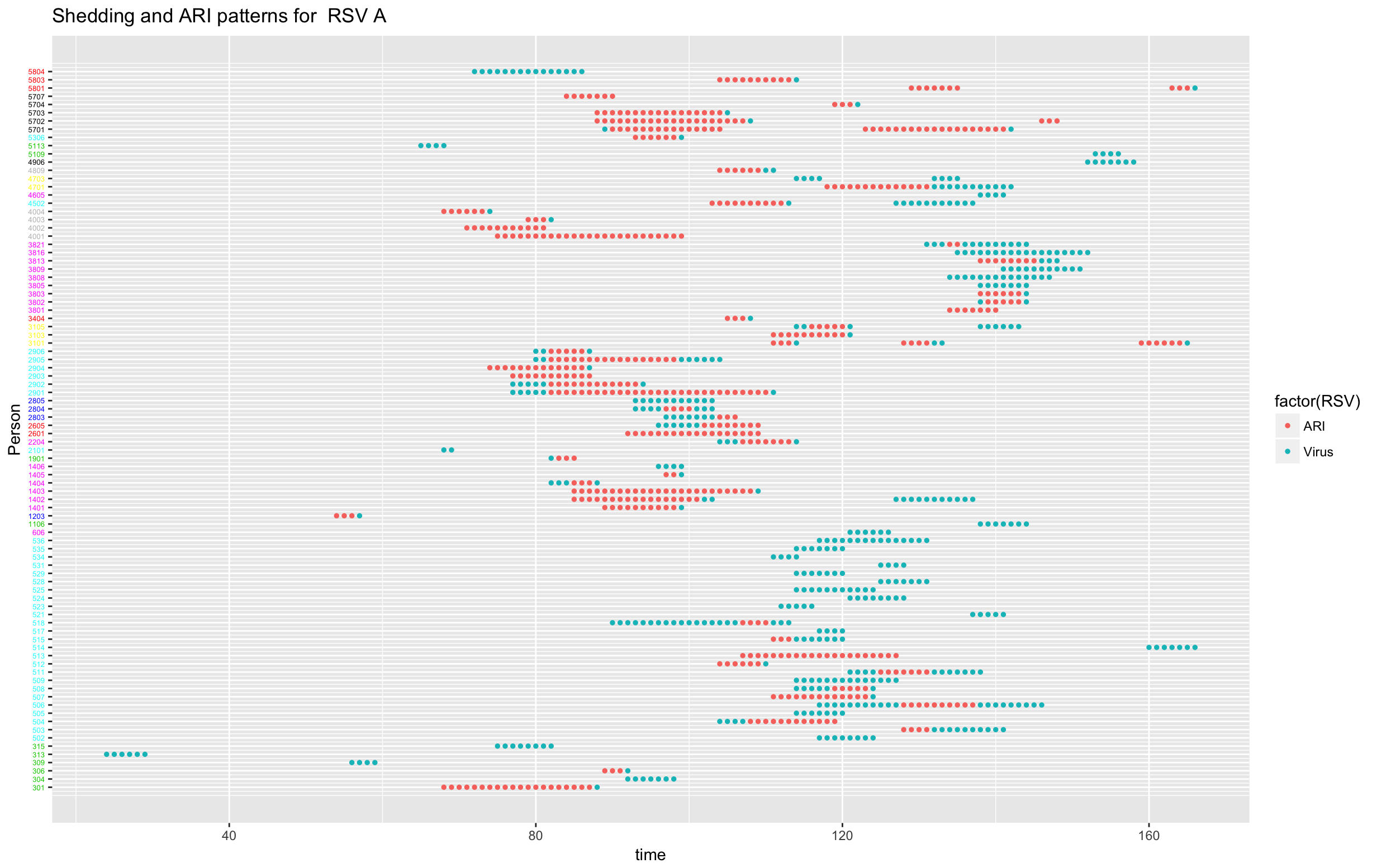


Figure A. 3: Shedding and ARI patterns for each of the 88 individuals who experienced at least one RSV A shedding episode. The y-axis shows the individuals with labels color-coded by household, time is on the x-axis with zero indicating the day before the first sample was collected. The green dots show virus shedding and orange dots show the virus shedding days that were accompanied by an ARI.


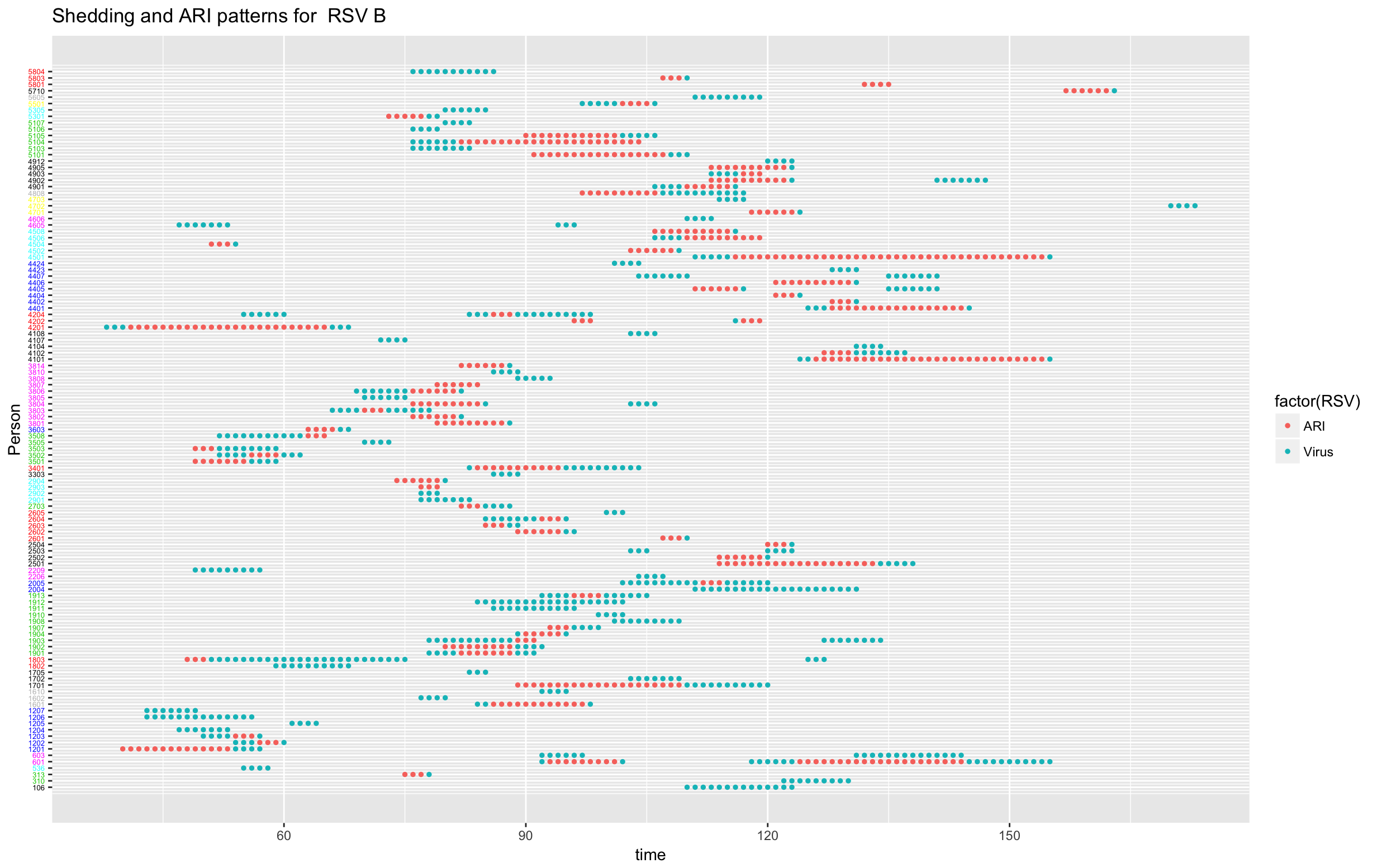


Figure A. 4: Shedding and ARI patterns for each of the 113 individuals who experienced at least one RSV B shedding episode. The y-axis shows the individuals with labels color-coded by household, time is on the x-axis with zero indicating the day before the first sample was collected. The green dots show virus shedding and orange dots show the virus shedding days that were accompanied by an ARI.

# Extra results

This section shows some additional results that are mentioned in the main text.

Three chains with different starting points were used to generate the parameter estimates. The trace plots are shown in Figure A.5. Chain 3 was run in three parts each with a length of 50000, 100000 and 100000 respectively. The starting point of the second part was the end point of the first part, and so on for the third part. This was done in an attempt to reduce total computation time. The Final results given after a burn-in of 80000 iterations exclude the re-start period seen between iteration 150000 and 175000. However, including it does not make a significant difference to the inferred posterior distributions.


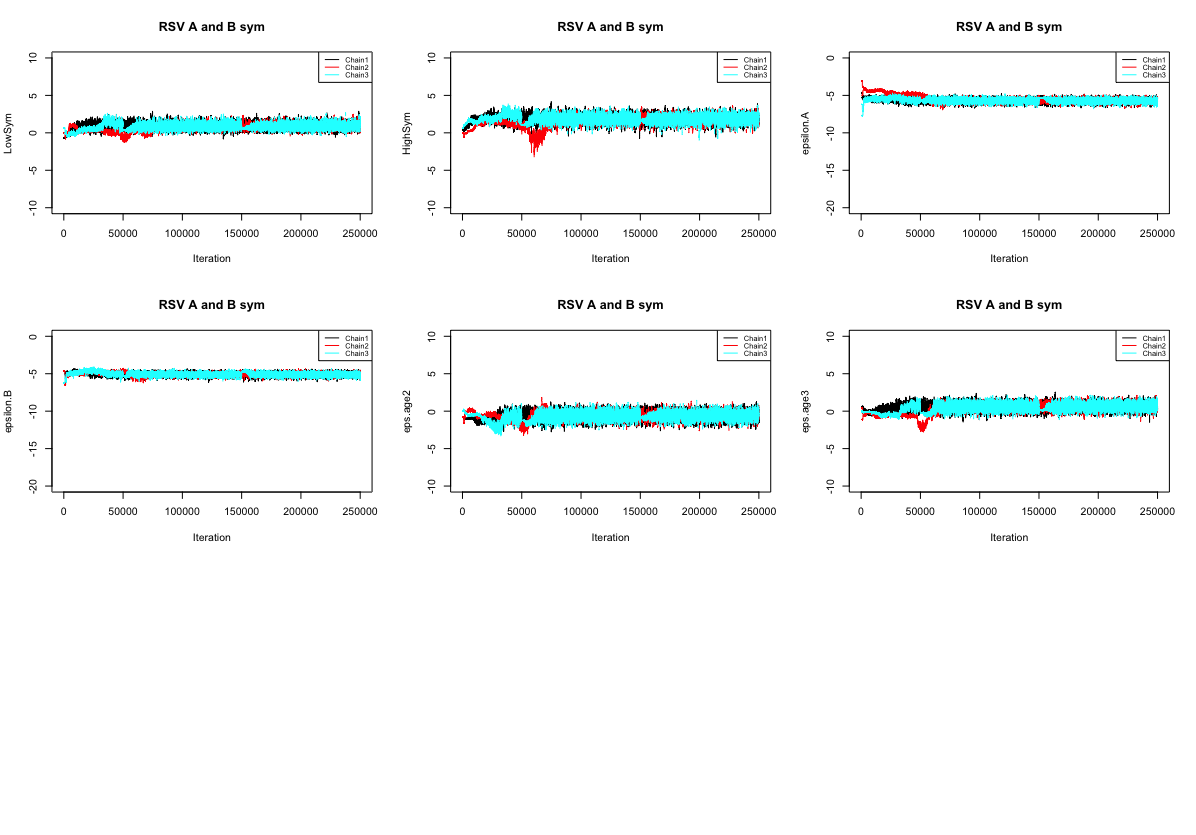

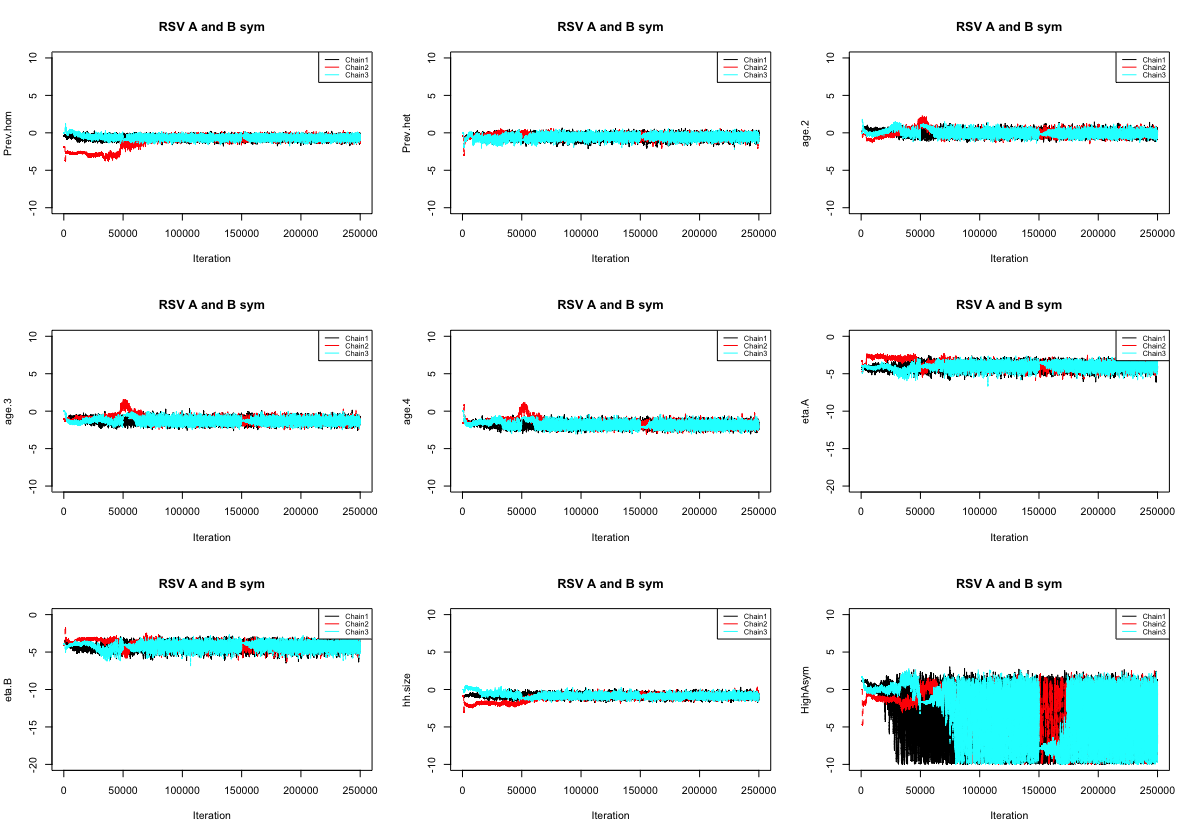


Figure A. 5: Trace plots showing convergence for the 15 parameters of interest. Three chains with different starting points were used.


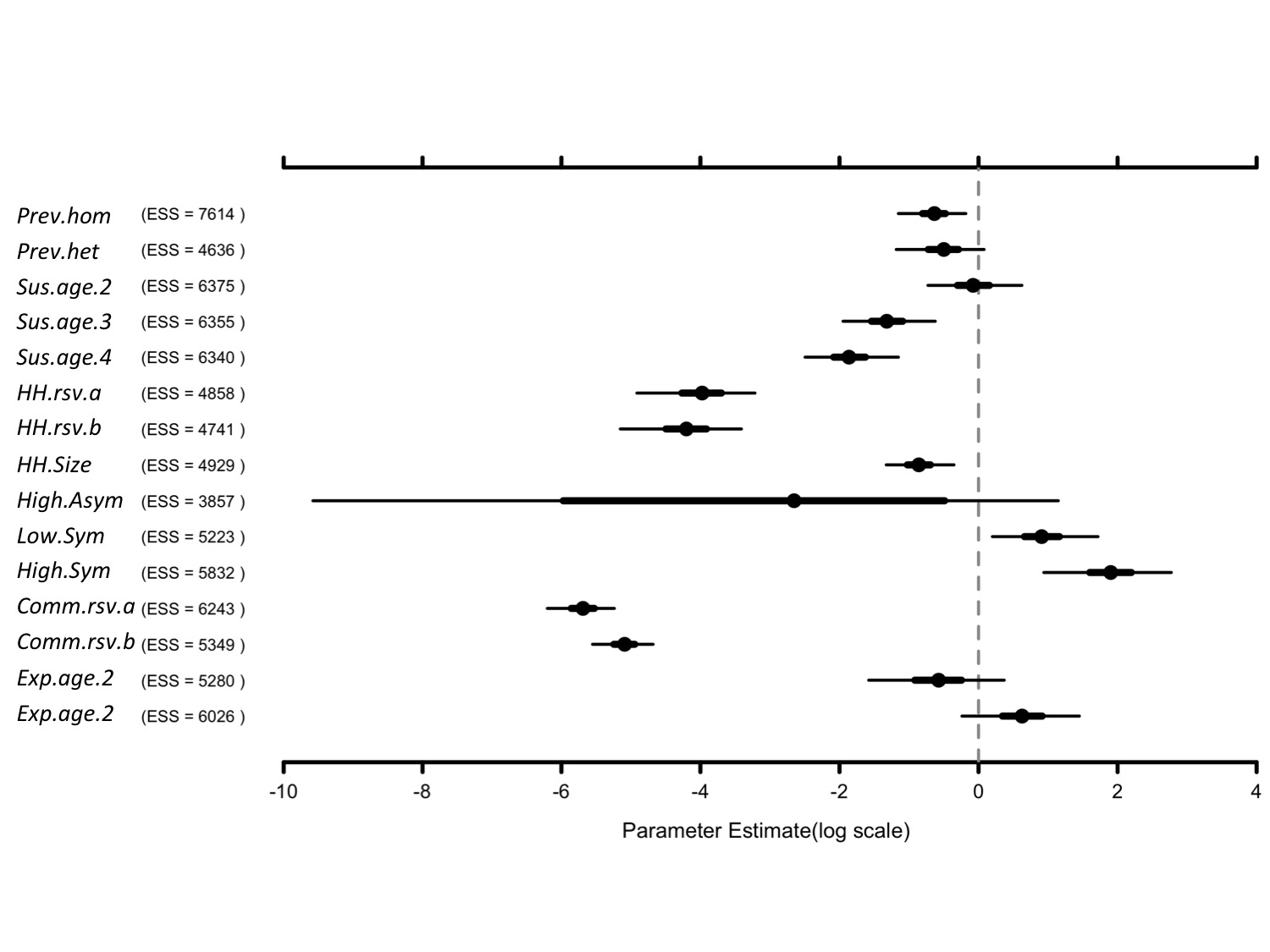


Figure A. 6: Caterpillar plot of estimated parameters. The 15 parameters estimated and their respective effective sample sizes are shown. Points represent posterior medians, the thick lines represent 50% credible region and the thin lines represent 95% credible region. Except *η_A_* and *η_B_* (within household transmission coefficients) *ε_A_*, and *ε_B_* (community transmission coefficients respectively) all the other parameters represent relative effects where a reference group exists. If a relative effect parameter is equal to 1(0 on the log scale) then the group it represents and the reference group are not different. Parameters where 50% credible interval overlaps with 0(dashed vertical line) are shown by open grey circles, where the 50% credible intervals do not overlap with 0 but the 95% credible interval does, filled grey circles show these parameters. If there is not overlap with 0, the circles are black and filled. ESS is the effective sample size


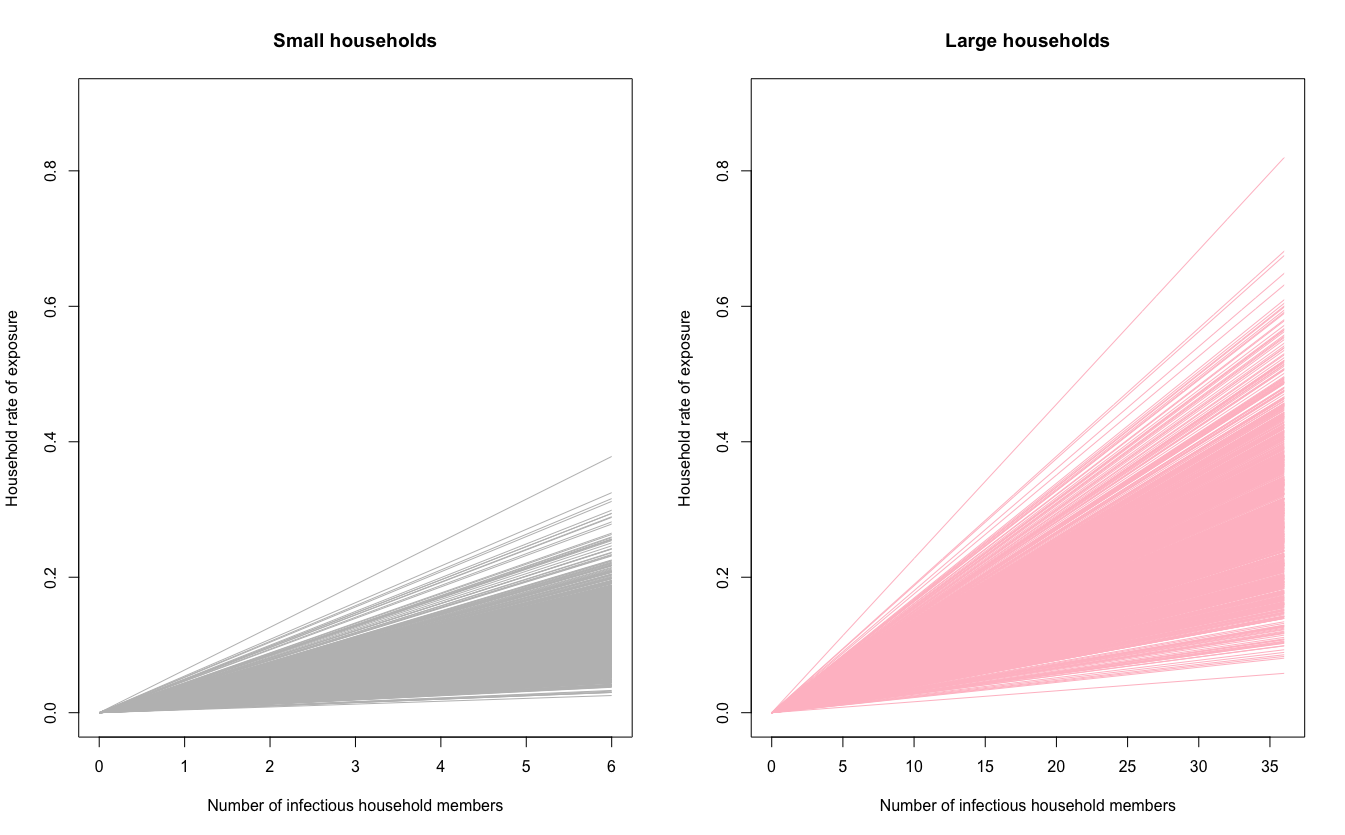


Figure A. 7: Comparing the total household rate of exposure $\left( \sum_{\boldsymbol{j\neq i}} \boldsymbol{HH.Risk}_{\boldsymbol{h,g,j}\boldsymbol{\to}\boldsymbol{i}}\left( \boldsymbol{t} \right) \right)$ between small and large households. Each line in the plots corresponds to parameter values in the posterior distribution such that each plot shows the 95% CrI of the linear relationship between total exposure rate and number of infectious house members.


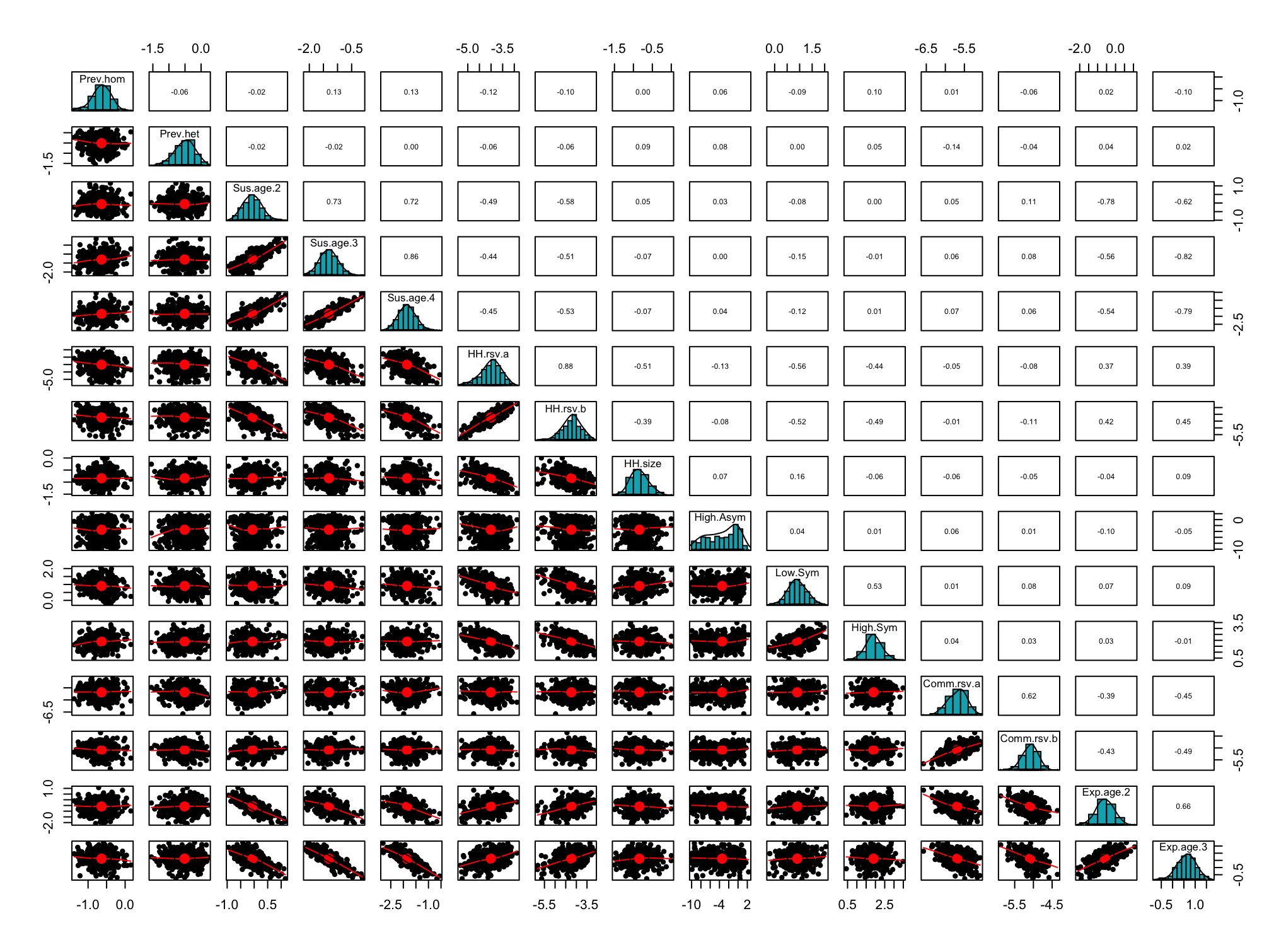


Figure A. 8: Correlation patterns of the different parameters obtained from fitting to the observed data.

Table A. 1: Results of fitting a reduced version of the model. We reduced the model such that there are no interactions between different RSV groups and refit the data in three additional ways: RSV A alone, RSV B alone and RSV with no distinction between groups.

| Parameter symbol | Parameter name | RSV A | | RSV B | | RSV |
| --- | --- | --- | --- | --- | --- | --- |
| $\boldsymbol{\phi}_{\boldsymbol{Y,hom}}$ | Prev.hom | 0.444 (0.0194, 0.963) | 0.547 (0.276, 0.983) | | 0.643 (0.423, 0.978) | |
| $\boldsymbol{\phi}_{\boldsymbol{X}\boldsymbol{2}}$ | Sus.aga.2 | 1.01 (0.436, 3.16) | 0.773 (0.263, 2.99) | | 0.919 (0.456, 2) | |
| $\boldsymbol{\phi}_{\boldsymbol{X}\boldsymbol{3}}$ | Sus.age.3 | 0.293 (0.134, 0.821) | 0.234 (0.0866, 0.859) | | 0.294 (0.154, 0.63) | |
| $\boldsymbol{\phi}_{\boldsymbol{X}\boldsymbol{4}}$ | Sus.age.4 | 0.187 (0.0829, 0.534) | 0.129 (0.047, 0.494) | | 0.159 (0.0811, 0.343) | |
| *η* | HH.rsv | 0.021 (0.00544, 0.0561) | 0.0101 (0.000453, 0.0343) | | 0.0133 (0.00457, 0.0311) | |
| $\boldsymbol{\phi}_{\boldsymbol{H}}$ | HH.size | 0.337 (0.184, 0.659) | 0.606 (0.285 1.52) | | 0.467 (0.277, 0.847) | |
| $\boldsymbol{\phi}_{\boldsymbol{I}\boldsymbol{2}}$ | High.Asym | 0.0401 (0.0000626, 1.72) | 0.243 (0.0000662, 18.1) | | 1.03 (0.000126, 6.36) | |
| $\boldsymbol{\phi}_{\boldsymbol{I}\boldsymbol{3}}$ | Low.Sym | 1.91 (0.711, 6.36) | 3.39 (1.20, 71.1) | | 2.17 (0.931, 5.93) | |
| $\boldsymbol{\phi}_{\boldsymbol{I}\boldsymbol{4}}$ | High.Sym | 7.28 (0.701, 25.4) | 6.31 (0.885, 158) | | 8.76 (3.72, 23.5) | |
| *ε* | Comm.rsv | 0.00328 (0.00159, 0.00594) | 0.006 (0.00339, 0.0096) | | 0.00939 (0.00588, 0.014) | |
| $\boldsymbol{\phi}_{\boldsymbol{E}\boldsymbol{2}}$ | Exp.age.2 | 0.335 (0.0745, 1.46) | 0.815 (0.16, 3.44) | | 0.574 (0.187, 1.65) | |
| $\boldsymbol{\phi}_{\boldsymbol{E}\boldsymbol{3}}$ | Exp.age.3 | 1.73 (0.548, 5.26) | 2.13 (0.484, 7.6) | | 1.81 (0.712, 4.4) | |

# Modification of the likelihood to establish the most likely infection source for every case.

The rate of exposure in the model is give as:

$$\lambda_{i,h,g}\left( t \right)=S_{i,g}(t)\left[ M_{i,h}(t)\sum_{j\neq i} {HH\_Risk}_{h,g,j\to i}\left( t \right)+ {Comm\_Risk}_{i,g}(t) \right]$$

This can be expanded to show all the variables and parameters as shown:

$$\lambda_{i,h,g}\left( t \right)=\exp\left( \phi_{Y,hist}(t)+ \phi_{X,age} \right)\left[ M_{i,h}(t)\sum_{j\neq i} \left( {\eta_{g}*\psi}_{H} * \psi_{I,inf} \right)+\left( \varepsilon_{g}*f_{g}\left( t \right)*\psi_{E,age} \right) \right] (1)$$

For a given case *i*, in order to be able to calculate the likelihood of infection from a particular source $\Omega_{i}$, either a sampled housemate or an unknown community source, we need to formulate the probability of transmission from said source at time *t*. This is given by:

$${Pr}_{\Omega_{i}\to i,h,g}\left( t \right)=\frac{\lambda_{\Omega_{i}\to i,h,g}\left( t \right)}{\lambda_{i,h,g}\left( t \right)} \left( 2 \right)$$

For $\Omega_{i}$ in the same household as *i*, the rate of exposure is given by

$$\lambda_{\Omega_{i}\to i,h,g}\left( t \right)=\exp\left( \phi_{Y,hist}\left( t \right)+ \phi_{X,age} \right)\left[ {{M_{i,h}\left( t \right)\eta}_{g}\psi}_{i,H}\psi_{\Omega_{i},I,inf}\left( t \right)M_{\Omega_{i},h}\left( t \right) \right]$$

For $\Omega_{i}$ an unknown source external to the household, the rate of exposure is given by

$$\lambda_{\Omega_{i}\to i,h,g}\left( t \right)=\exp\left( \phi_{Y,hist}\left( t \right)+ \phi_{X,age} \right)\left[ \varepsilon_{g}*f_{g}\left( t \right)*\psi_{E,age} \right]$$

*The likelihood function*

The probability given in (2) is calculated for a time point *t* = exposure time of individual *i,* $t_{i}^{E}$. This is not observed in the data, however, given our assumption on the latency duration, we can define a 6-day window of possibility. If case *i* had a shedding onset at time $T_{i}^{O}$, then the window for transmission is from day $\left( T_{i}^{O}-5 \right)$ to $\left( T_{i}^{O}-0 \right)$. For each day in the window, potential sources are identified based on shedding status and for each combination of infection source $\Omega_{i}$ and exposure date $t_{i}^{E}$, the likelihood is calculated using the formula below:

$$L\left( \varphi| \left\{ T_{i}^{o},t_{i}^{E},\Omega_{i} \right\} \right)=\left( 1-e^{-\lambda_{i,g,h}\left( t_{i}^{E} \right)} \right)*\left( \prod_{t_{i}{\neq t}_{i}^{E}} e^{-\lambda_{i,g,h}\left( t_{i} \right)} \right)*\left( \theta_{l}\left( T_{i}^{o}-t_{i}^{E} \right) \right)*\left( \frac{\lambda_{\Omega_{i}\to i,h,g}\left( t_{i}^{E} \right)}{\lambda_{i,h,g}\left( t_{i}^{E} \right)} \right)$$

The first part of the product is the probability of infection at time $t_{i}^{E}$, the second part is the probability of escaping infection at any time $t_{i}{\neq t}_{i}^{E}$, the third is the probability of a latency duration of length $\left( T_{i}^{o}-t_{i}^{E} \right)$and the last term is the probability of transmission from source $\Omega_{i}$ to *i*.

Given the likelihood, the highest-probability-source is chosen as the infection source that give the highest value of the likelihood.

# Model validation

This is a two part process: first we check if the parameters estimated can reproduce the results (or something similar) by simulation; then we check if given simulated data, we can re-estimate parameters that are similar to the ones used to simulate the data. This process is illustrated in the flow chart below.

Figure A. 9: Flow chart showing validation process

Given a set of parameter values, the simulation pseudo code per simulation is as follows:

1. Initiate system such that everyone one is susceptible to RSV A and RSV B.
2. At every time step keep track of:
   1. Susceptibility status of every individual
   2. Exposure status
   3. Infectious status (viral load and infectivity group)
   4. Infection history
3. At every time step:
   1. Determine number of transmission events using

$$\Delta E=Poisson\left( \sum_{i\in C_{E}} \left( 1-e^{-{rate}_{E,i}} \right) \right)$$

ΔE = number of events of type *E* at a given time point

*C_E_* = set of all individuals capable of experiencing event *E*.

*rate_E,i_* = rate of occurrence of event E on person *i*.

- 1. Determine who experiences each event. For a given event, order individuals capable of experiencing the event. For a given person *p* to experience the event, the following inequality has to be satisfied.

$$\sum_{i=1}^{i\leq p-1} P_{E,i} <\left( RAND \times\sum_{i \in C_{E}} P_{E,i} \right) \leq\sum_{i=1}^{i\leq p} P_{E,i}$$

Where $P_{E,i}=1-e^{-{rate}_{E,i}}$ = probability of person *i* experiencing event E. *RAND* = a random number between (but not including) 0 and 1. This is illustrated in the figure below.


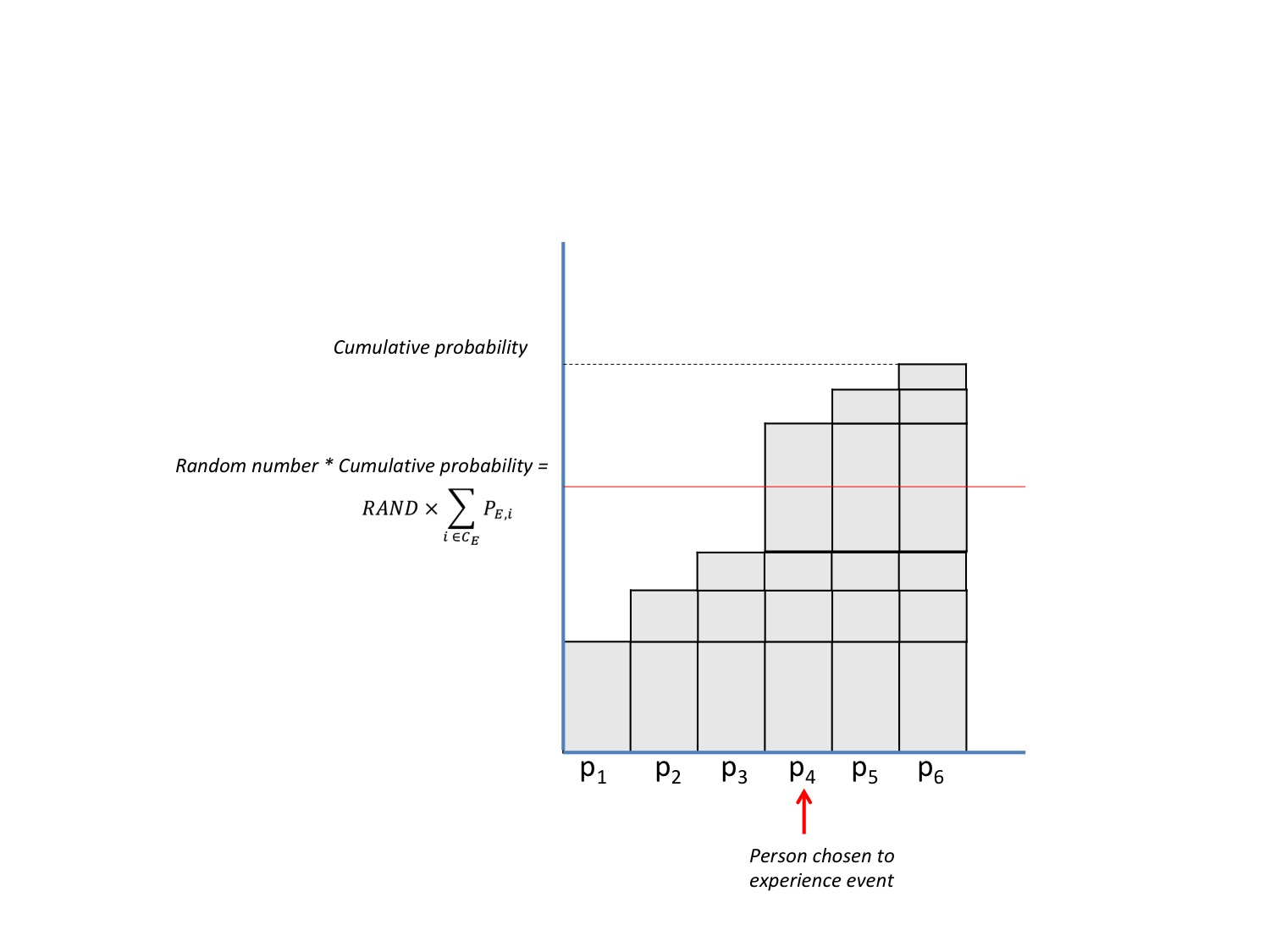


Repeat this until the required number of events

- 1. For each individual experiencing a transmission event, assign a latency duration and shedding profile by sampling from the relevant empirical distributions. The empirical latency distribution is the same as was used in estimating the parameters, and is homogeneous for every individual. The shedding profiles are grouped by age in the following 4 groups <1,1-5, 5-15 and ≥15 years (see Figure A. 10 and Figure A. 11 for age grouped shedding profiles). An assigned shedding profile is a combination of duration of shedding, viral loads and symptom status. Once latency durations and shedding profiles have been assigned, the state variables for each individual are updated accordingly.
  2. Update rate of exposure.

The rate of exposure/transmission for susceptible individuals changes according to

$$\lambda_{ihg}\left( t \right)=exp(\phi_{X}X_{i}+\phi_{Yg}Y_{ig}(t))\left[ M_{ih}(t){\eta_{g} \phi}_{H}H_{i}\sum_{j\neq i} {\phi_{I}I}_{jhg}\left( t \right)+ \phi_{E}E_{i}\varepsilon_{g}f_{g}(t) \right]$$

Figure A. 10 and Figure A. 11 show the shedding profiles as observed from the data for RSV A and B, clustered by age and symptom status.


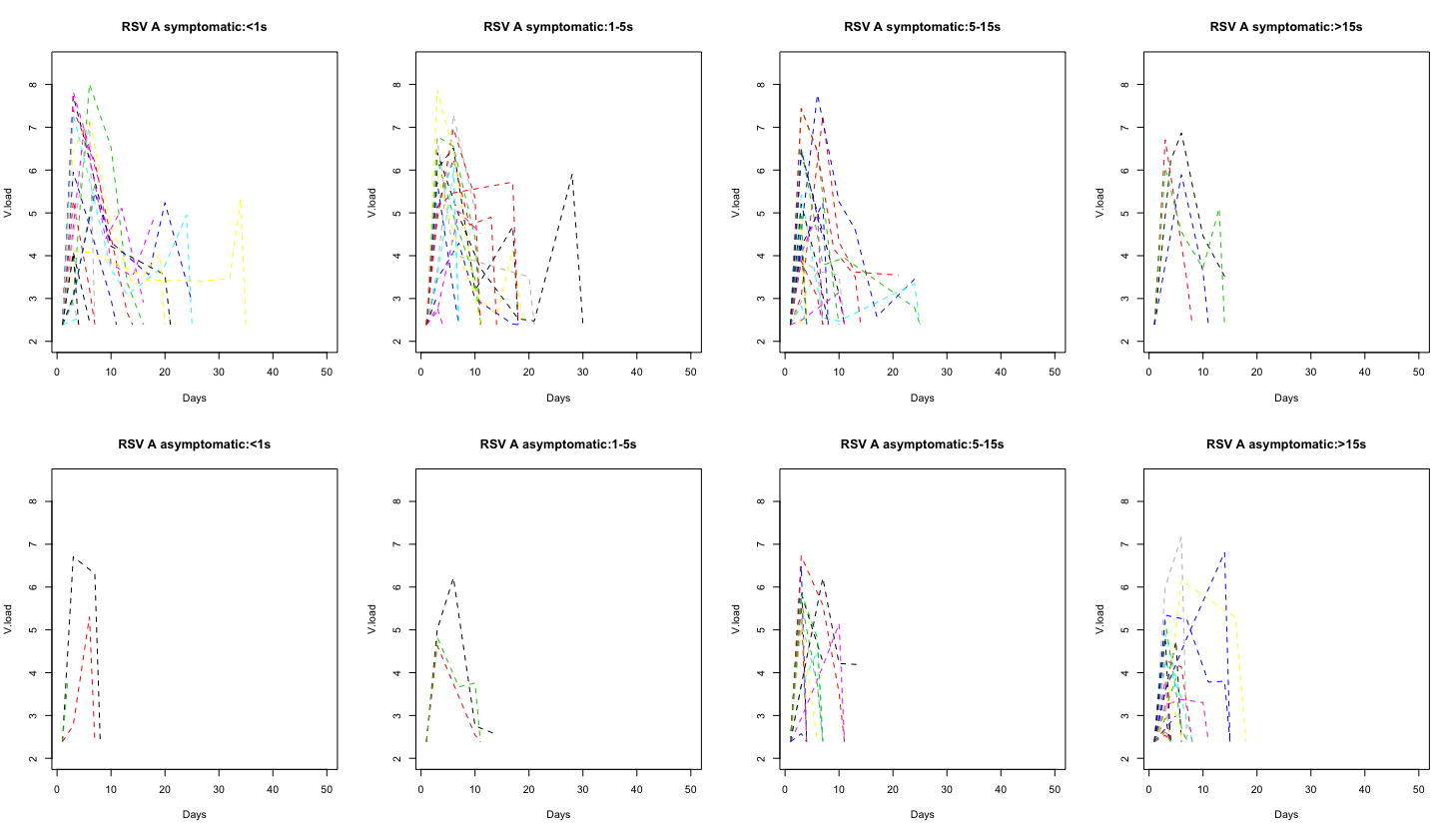


Figure A. 10: RSV A shedding profiles as observed. Each figure shows the viral loads on different days of shedding for each infection episode observed. The top row shows profiles for symptomatic RSV A shedding by age group in years, the bottom row shows profiles for asymptomatic RSV A shedding by age group in years.


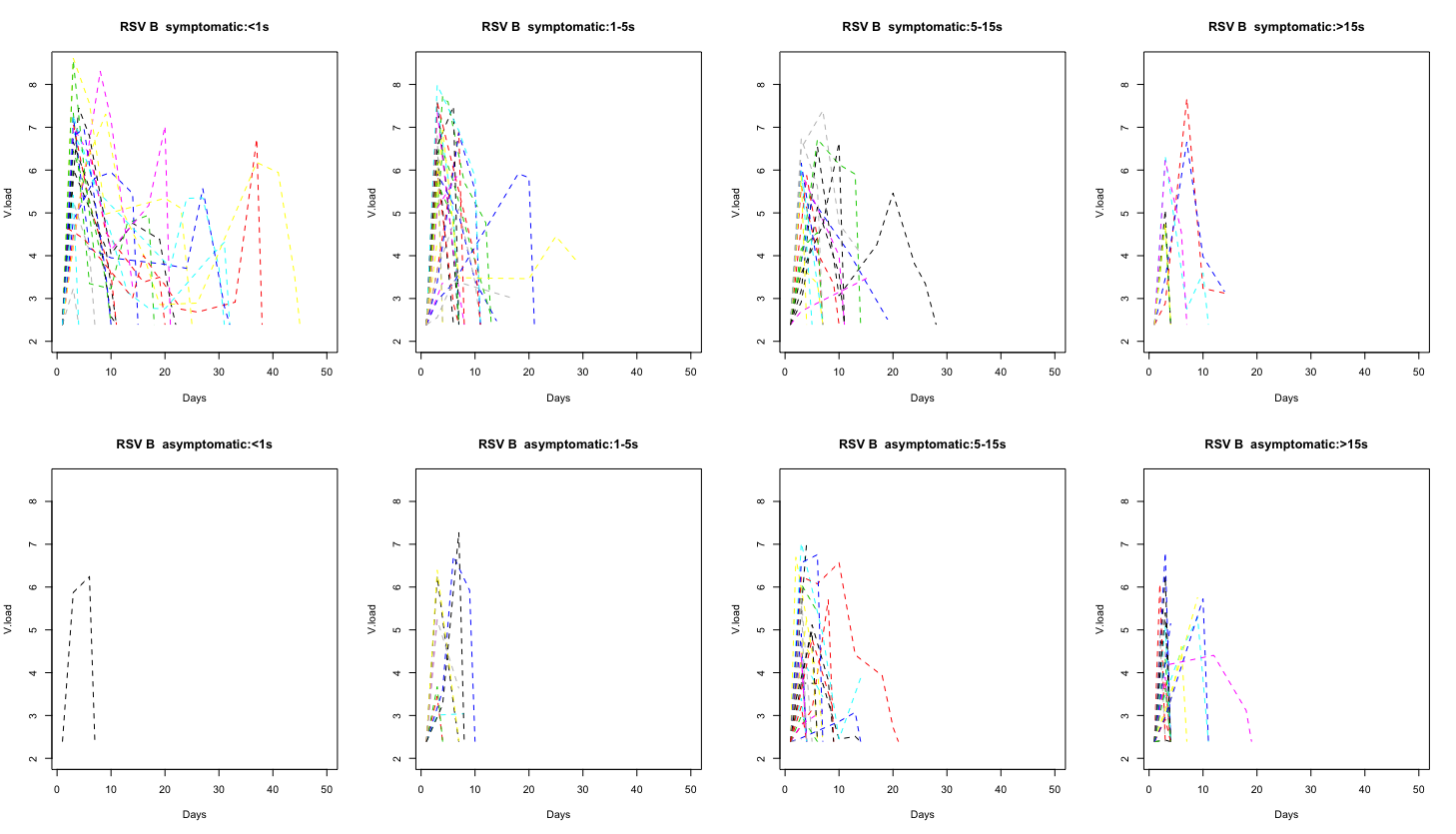


Figure A. 11: RSV B shedding profiles as observed. Each figure shows the viral loads on different days of shedding for each infection episode observed. The top row shows profiles for symptomatic RSV B shedding by age group in years, the bottom row shows profiles for asymptomatic RSV B shedding by age group in years.

We sampled 5 sets of parameters (dependent sampling to maintain the correlations observed) and for each set simulated 200 epidemics to compare to the data. The sampled parameters relative to the posterior distribution are shown in Figure A. 12. In addition to looking at the projected epidemics, we also look at the following outcome measures to make comparisons:

- Total number of individuals infected

- Total number of households infected

- Proportion of individuals with repeat infections

- Timing of epidemic peak

Figure A. 13 and Figure 4 in the main text show the results of the simulations relative to the observed data.


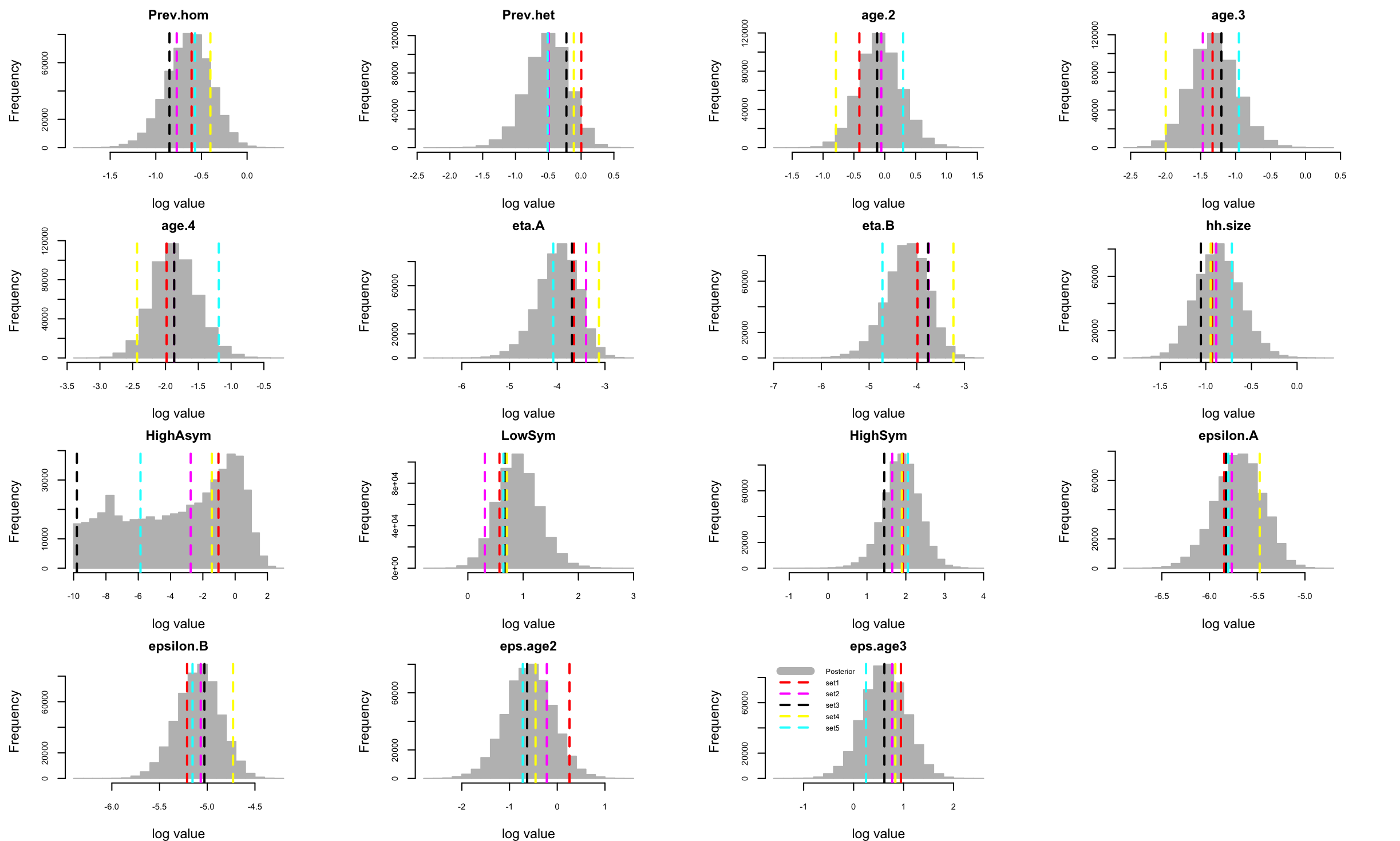


Figure A. 12: Histograms of the posterior distributions with vertical lines showing sample sets that were used in simulation. Each panel shows histograms of different parameters in grey. Red dashed lines show the value of the parameter in set1, dark pink shows set2, black set3, yellow set4 and blue set5.


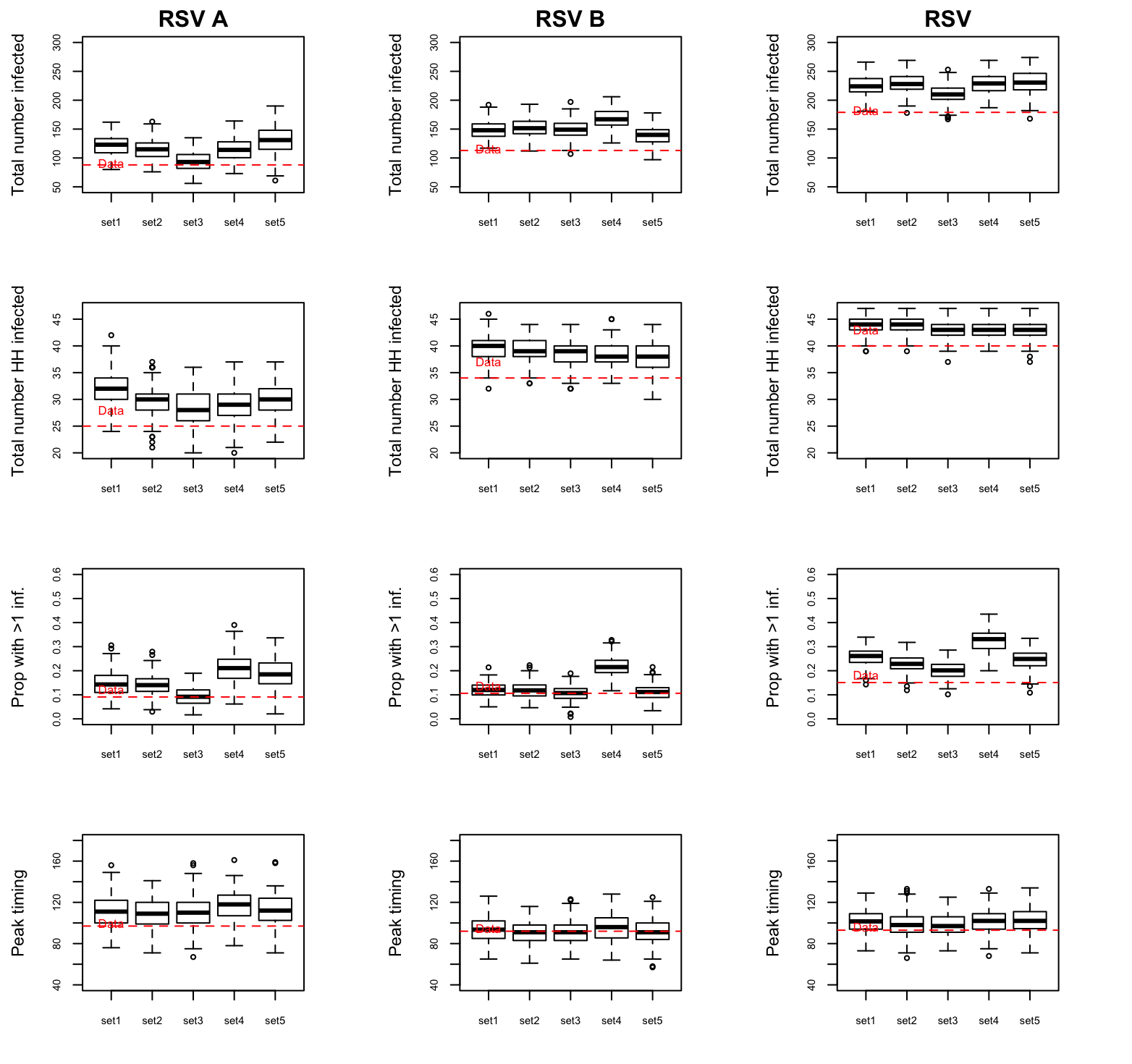


Figure A. 13: Outcome measures from simulated data when using different sets of parameters drawn from the posterior estimated from the observed data. Each box and whisker plot is the distribution of the specific outcome measure from 200 simulations run from a single sampled data set.

To check if the model can re-estimate known parameter values, we simulated an epidemic and compared the re-estimated densities to the densities given by using the observed data. Table A. 2 gives the values of the parameters used to simulate the epidemic, Figure A. 14 compares the real and simulated epidemics and Figure A. 15 compares the original and re-estimated parameter densities.

Table A. 2: Parameter set used to simulate an epidemic

| Parameter symbol | Parameter name | Set1 |
| --- | --- | --- |
| $\boldsymbol{\phi}_{\boldsymbol{Y,h}\boldsymbol{om}}$ | Prev.hom | 0.544 |
| $\boldsymbol{\phi}_{\boldsymbol{Y,het}}$ | Prev.het | 1 |
| $\boldsymbol{\phi}_{\boldsymbol{X}\boldsymbol{2}}$ | Age.2 | 0.662 |
| $\boldsymbol{\phi}_{\boldsymbol{X}\boldsymbol{3}}$ | Age.3 | 0.265 |
| $\boldsymbol{\phi}_{\boldsymbol{X}\boldsymbol{4}}$ | Age.4 | 0.138 |
| *η_A_* | Eta.A | 0.026 |
| *η_B_* | Eta.B | 0.0186 |
| $\boldsymbol{\psi}_{\boldsymbol{H}}$ | hh.size | 0.394 |
| $\boldsymbol{\psi}_{\boldsymbol{I}\boldsymbol{2}}$ | HighAsym | 0.356 |
| $\boldsymbol{\psi}_{\boldsymbol{I}\boldsymbol{3}}$ | LowSym | 1.77 |
| $\boldsymbol{\psi}_{\boldsymbol{I}\boldsymbol{4}}$ | HighSym | 6.85 |
| *ε_A_* | Epsilon.a | 0.00289 |
| *ε_B_* | Epsilon.b | 0.00545 |
| $\boldsymbol{\psi}_{\boldsymbol{E}\boldsymbol{2}}$ | Eps.age2 | 1.29 |
| $\boldsymbol{\psi}_{\boldsymbol{E}\boldsymbol{3}}$ | Eps.age3 | 2.57 |


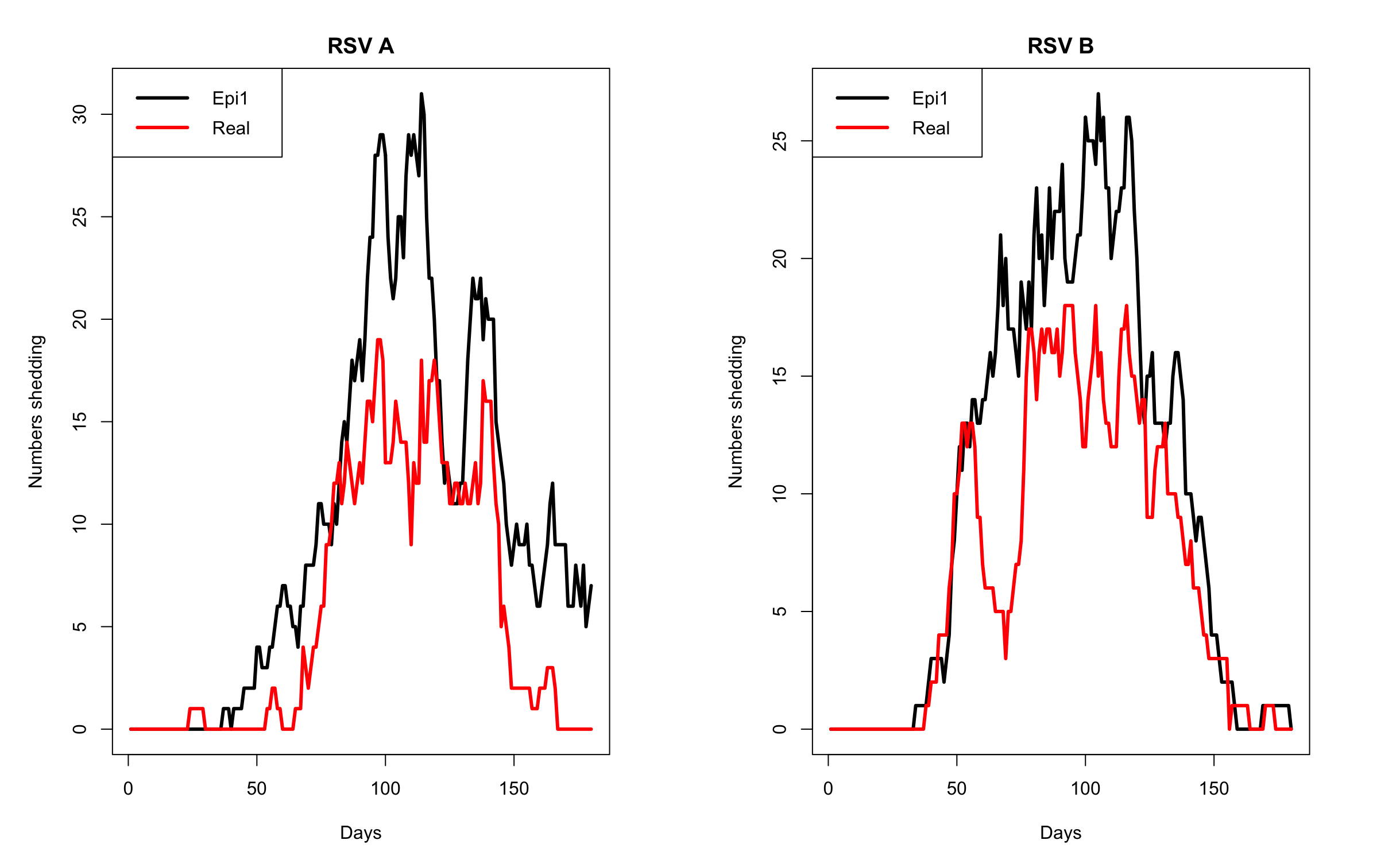


Figure A. 14: Comparing the real (red lines) and simulated( black lines) epidemics


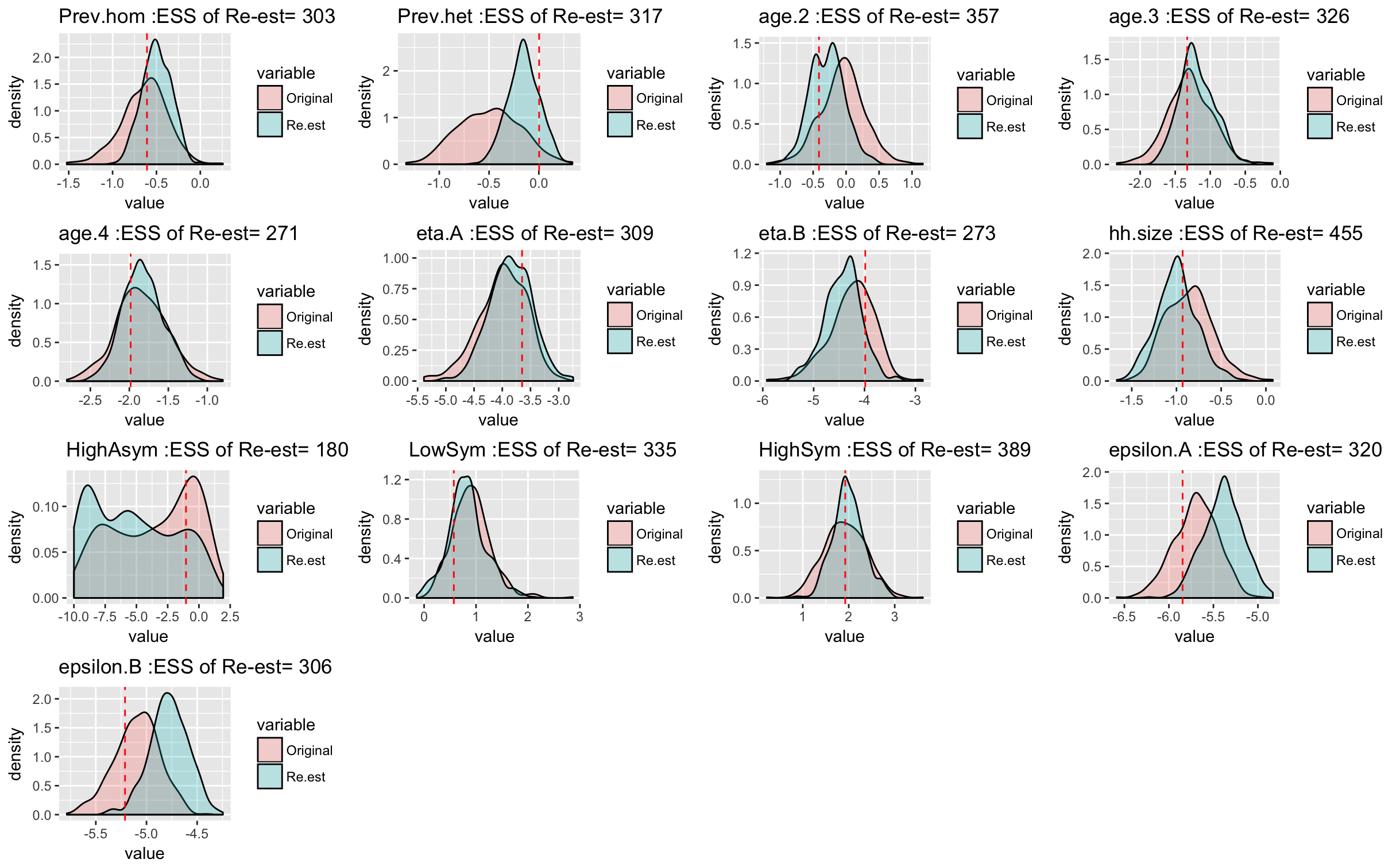


Figure A. 15: Comparing the posterior densities obtained from using the observed data to those from using the simulated data. The posterior densities from using real data are shown in red while the ones from simulated data are shown in blue. The dashed red line shows the value of the parameter used to simulate the epidemic. ESS is the effective sample size.

The re-estimated distributions capture the parameter used to simulate the epidemic and in general fall within the ranges of the original distributions obtained from the real data.

# Sensitivity analysis

We check if our results were sensitive to the background community density function by exploring 3 additional function forms. The results are presented in the following three figures. Option1 shows the density curves used in the main analysis, Options 2,3 and 4 show the curves used in the sensitivity analysis.


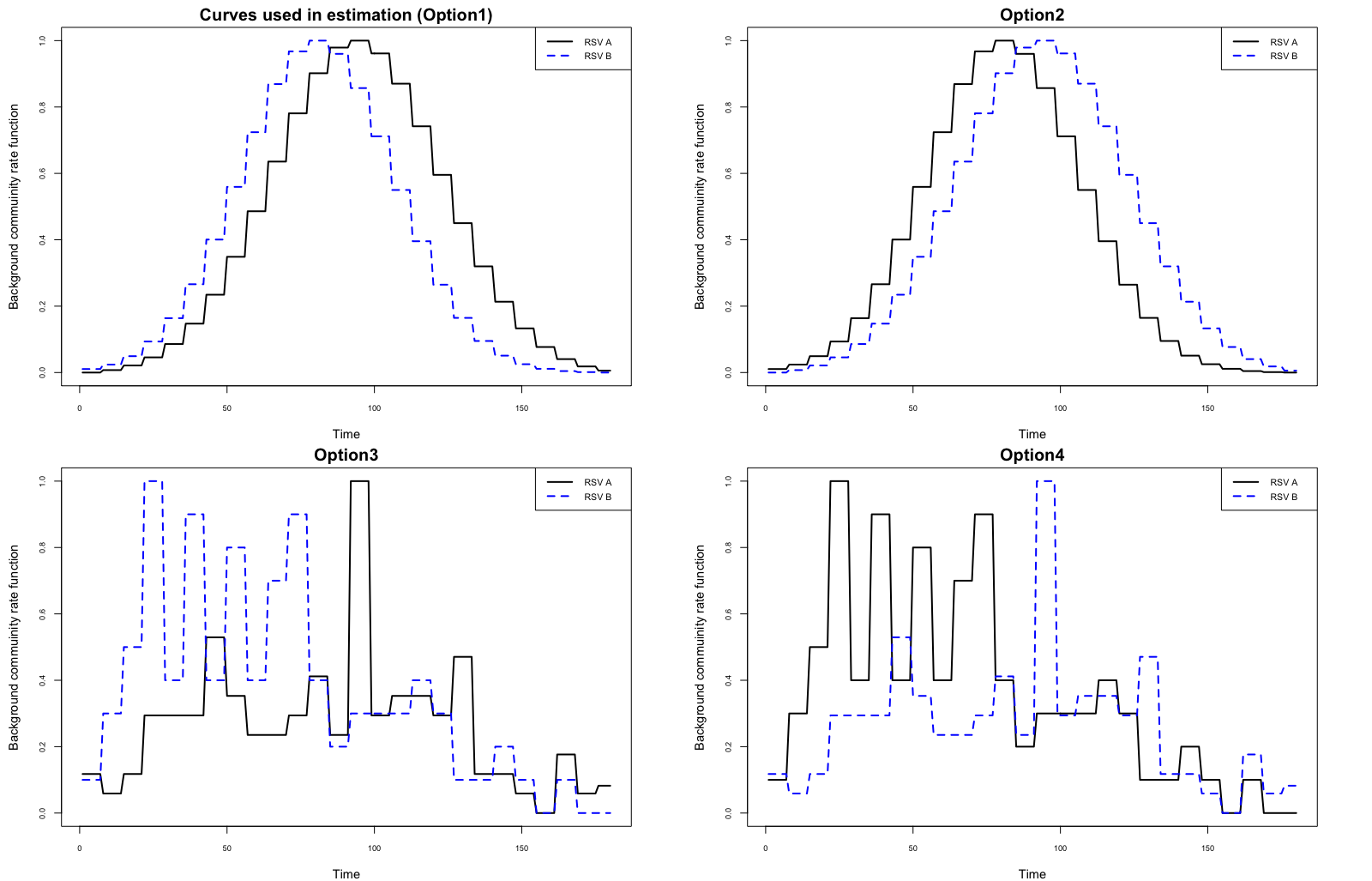

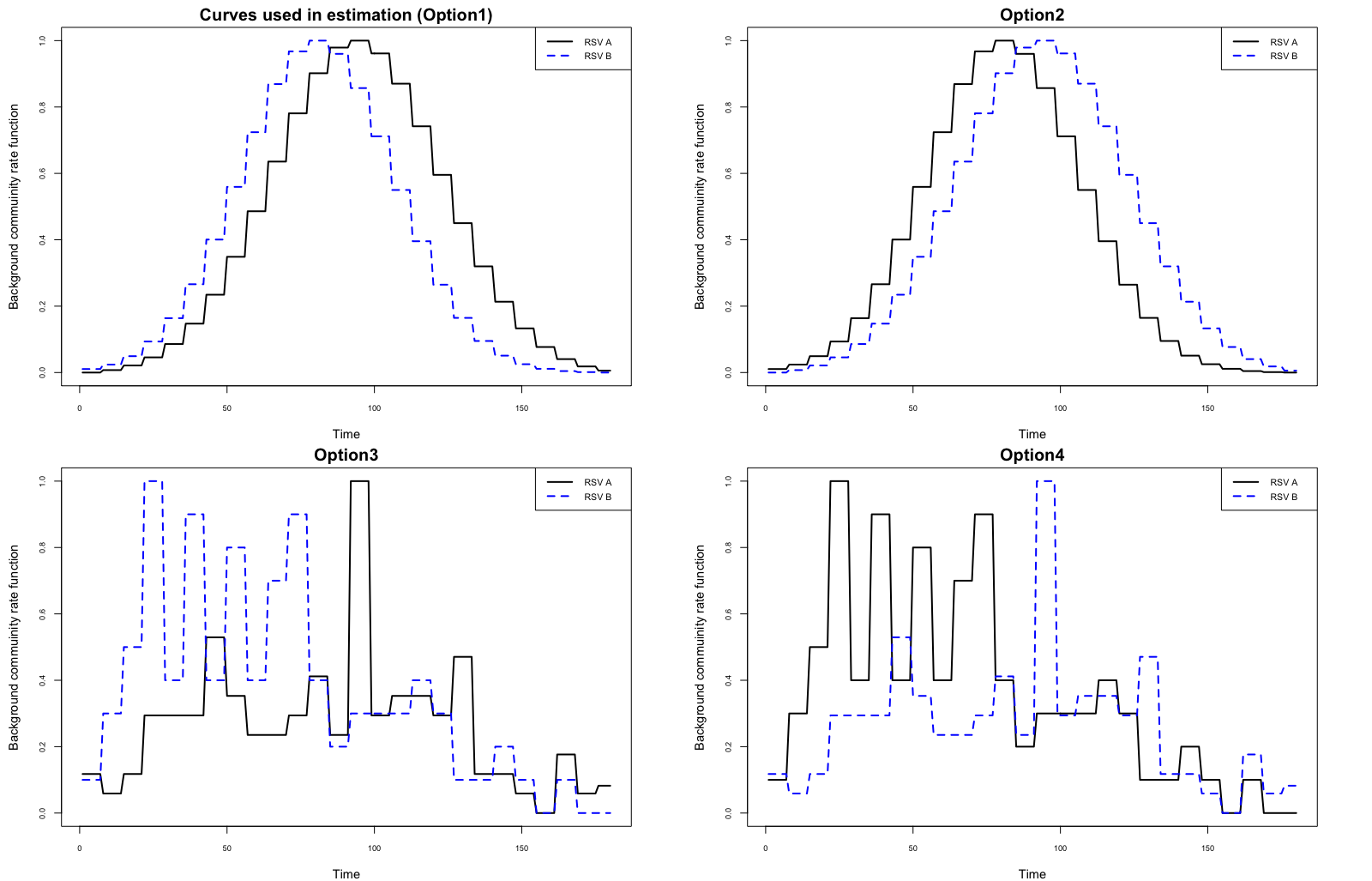

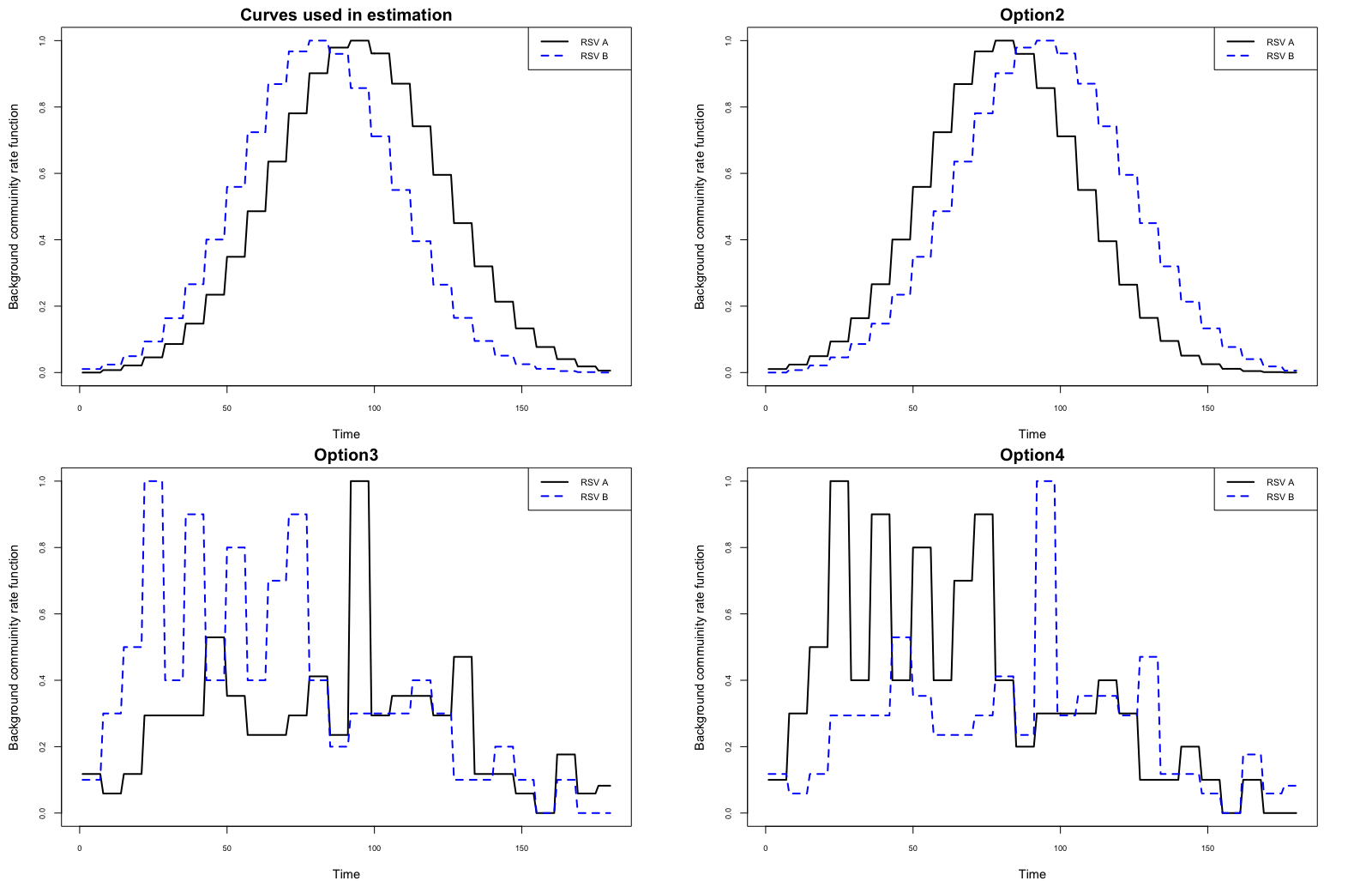

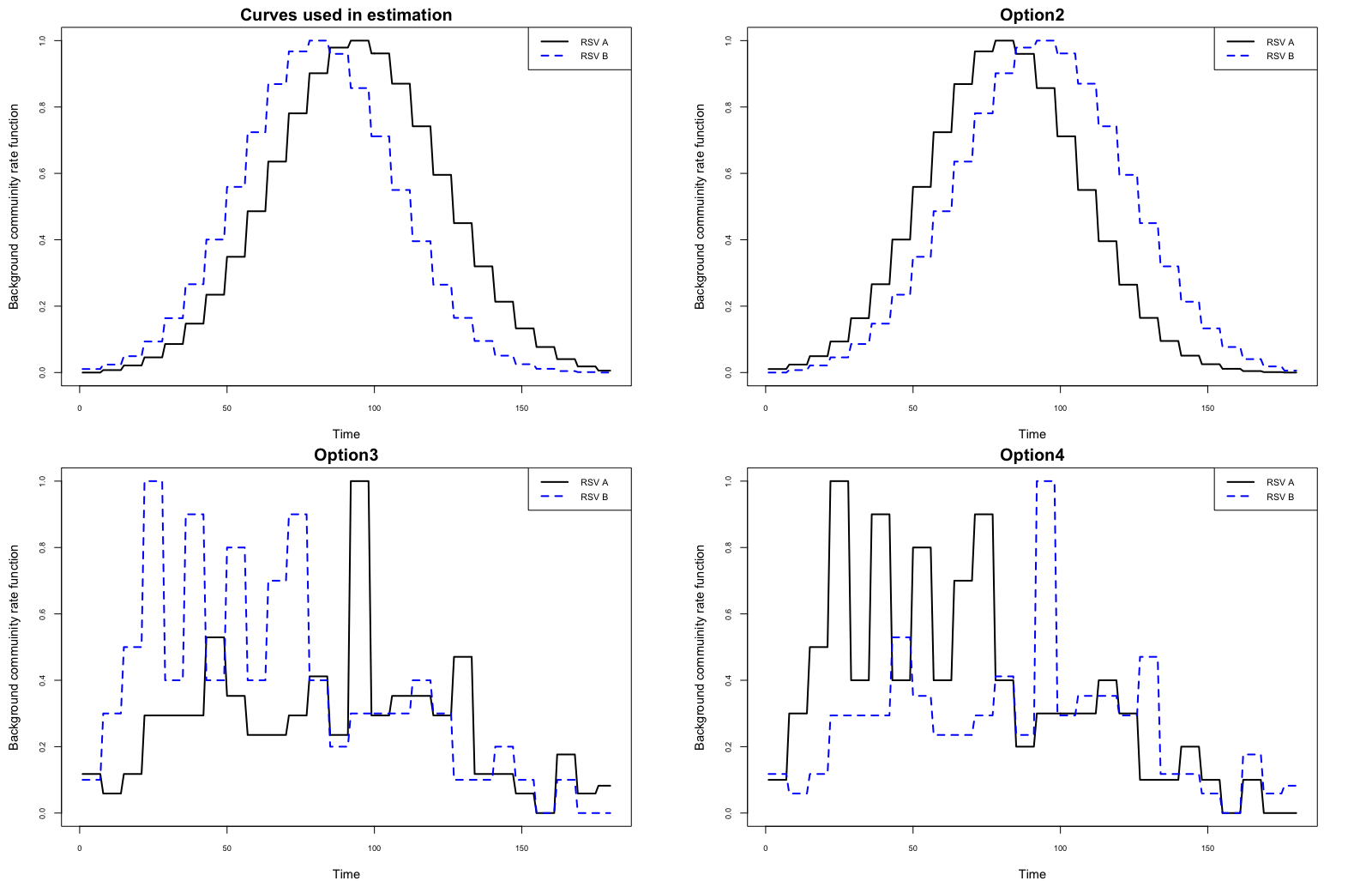

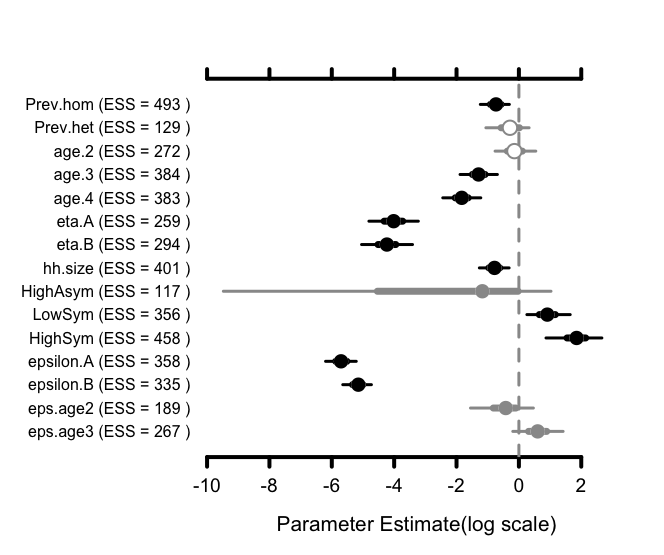

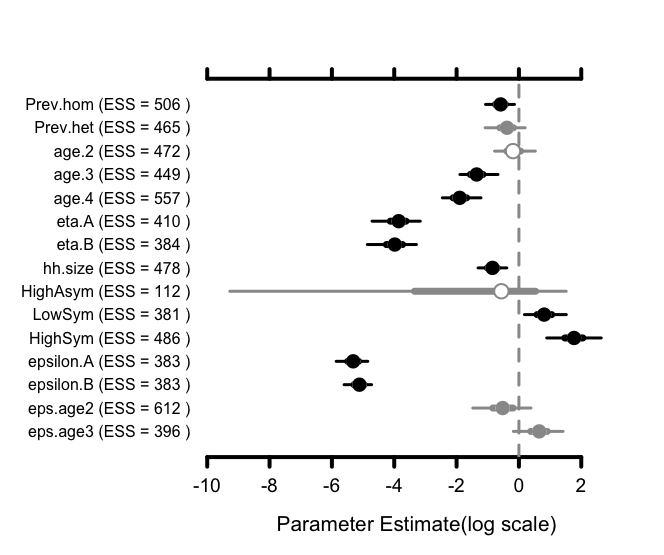

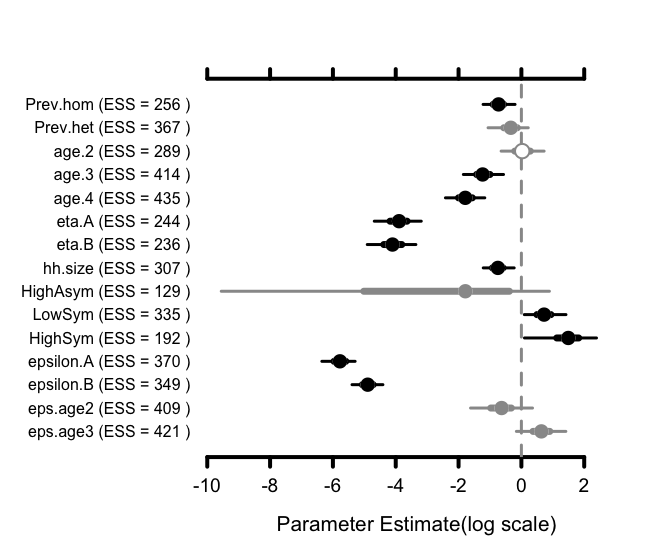


Figure A. 16: Using different density functions for the background community rate and comparing results. The left side shows the density functions for RSV A and RSV B, the right side shows the re-estimated parameters after 20,000 iterations.


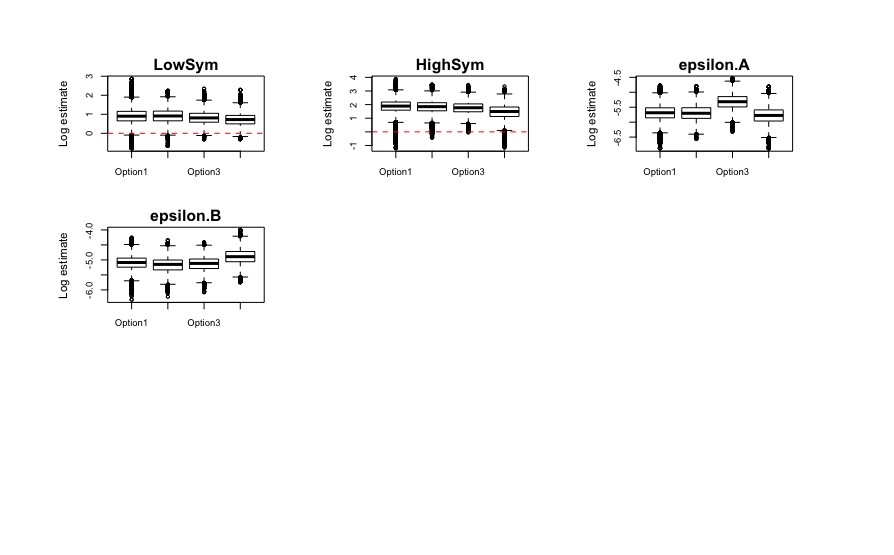

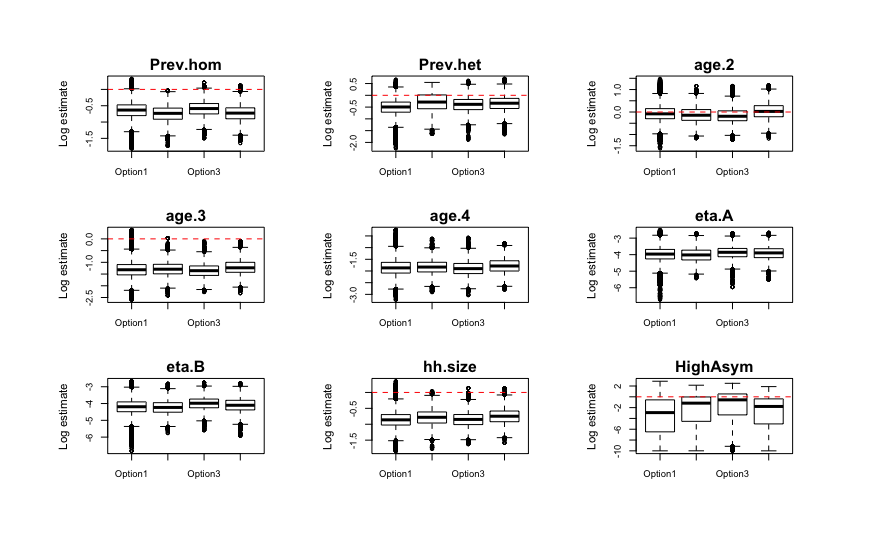


Figure A. 17: Using different density functions for the background community rate and comparing results. Box plots comparing the estimated parameters.


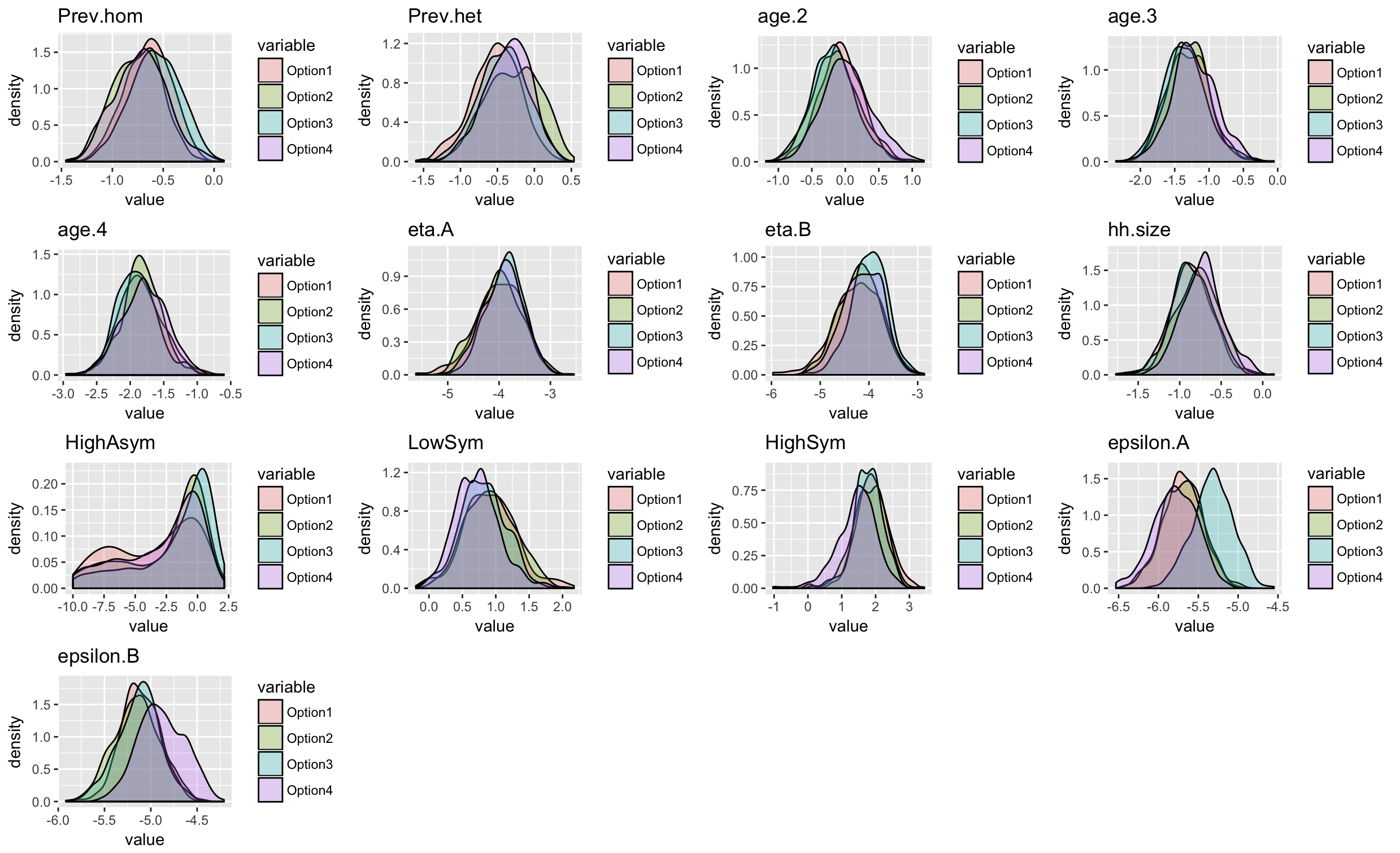


Figure A. 18: Using different density functions for the background community rate and comparing results. Density plots comparing the estimated parameters.

We also looked at the distribution of cases by household size. In Figure A. 19 we see that RSV A got into the largest household and infected significantly more people that RSV B. Looking at Figure A. 20, it seems that all but one RSV A case were probably part of a single outbreak (based on perceived temporal distance). To check if this could be the reason for the difference in within household transmission coefficient estimated, we removed data from the largest household (HH5) and re-estimated the parameters. The results of this are show in Figure A. 21 and Figure A. 22. The slight difference between the RSV groups in the within household transmission parameter is still present.


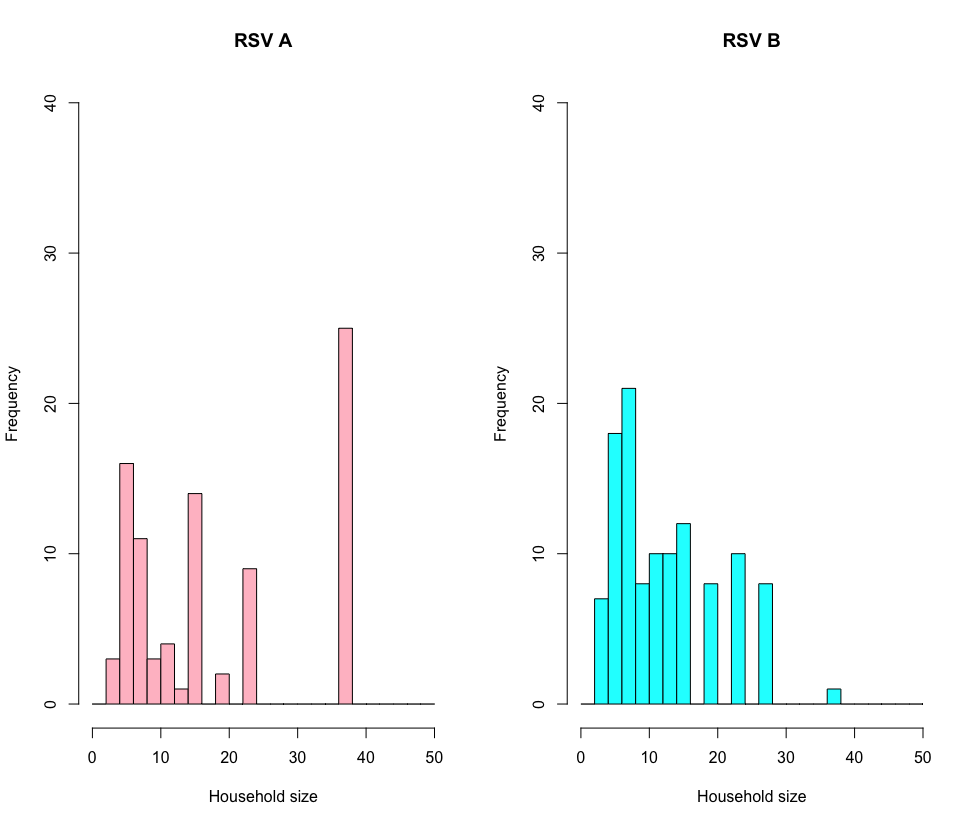


Figure A. 19: Frequency distributions of RSV A and RSV B infections by household size

Figure A. 20: Infection patterns in HH5


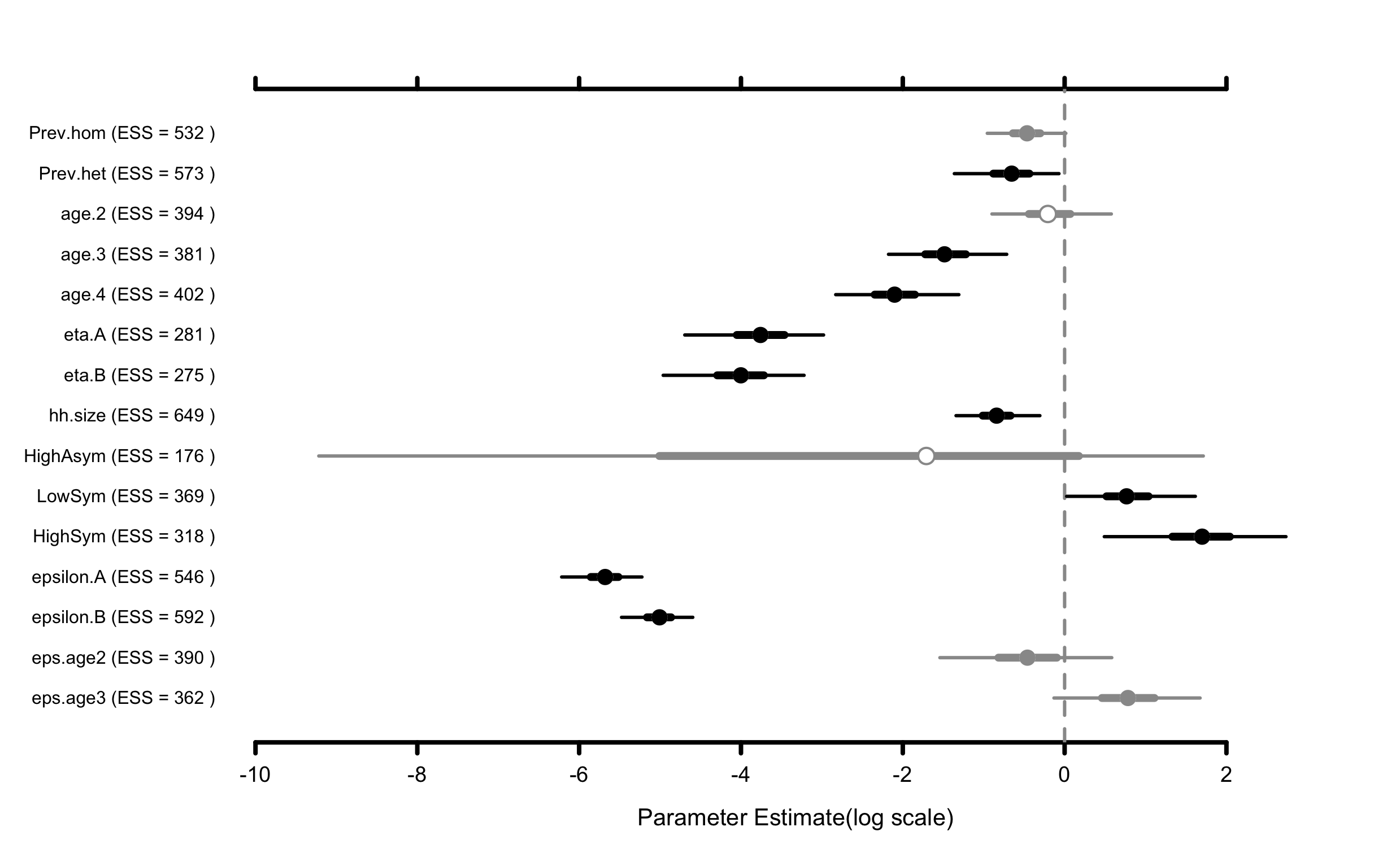


Figure A. 21: Caterpillar plot showing results obtained when household 5 data was removed from the set.


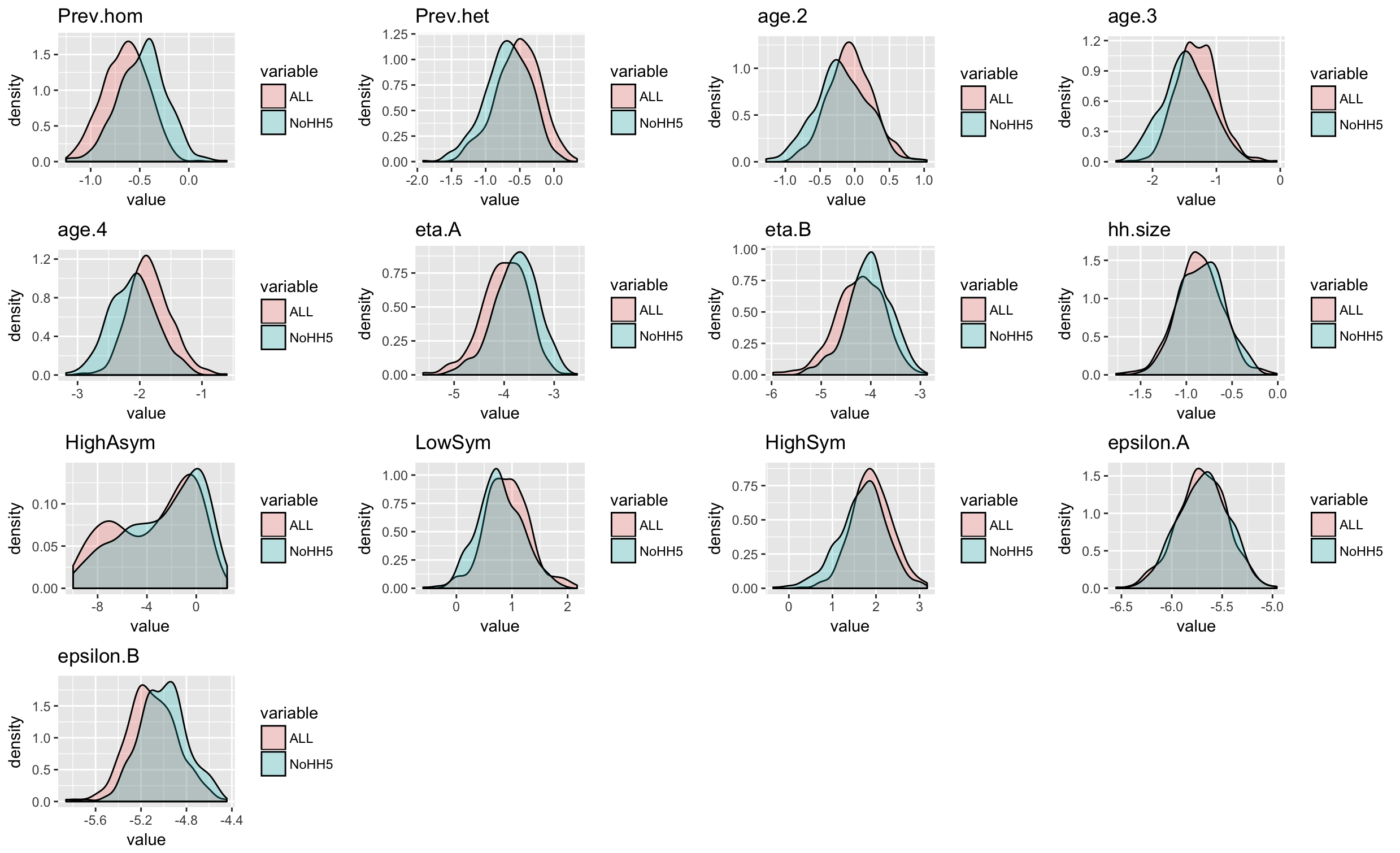


Figure A. 22: Comparing densities of parameters estimates obtained when using all the data (light red) to densities obtained when using data without household 5 (light blue).

# Checking the contribution of symptomatic and asymptomatic individuals

In this section we show the results of simulations where infectiousness was altered, Figure A. 23, and parameter estimation where only a subset of the data was used Figure A. 24. For the simulation we compare three scenarios: Infectiousness of symptomatics and asymptomatics as given in the model parameters presented in Table 3; Infectiousness of the symptomatic individuals is reduced to match that of asymptomatic individuals (this is done so as to get an idea of what the effect of a vaccine that reduces symptoms would be); Infectiousness of asymptomatic individuals is assumed to be 0 such that they cannot transmit (this is done so as to get an idea of the contribution of asymptomatic infections to transmission). For each scenario, 10000 simulations were used based on sampling 100 different parameter(and making the modifications necessary for scenario 2 and 3) sets and for each set simulation 100 epidemics.

Figure A. 23: Densities comparing the relative total incidence, by RSV group and age group, when the infectiousness of symptomatic individuals is altered or when the infectiousness of asymptomatic individuals is removed. The black line shows the distribution of total number of people infected from 10000 simulations for estimated (unaltered) parameters scenario where symptomatic individuals are more infectious than asymptomatic. The red line shows the case when where the parameters used in simulation have been altered to force symptomatic individuals to be as infectious as asymptomatic individual (i.e. reduced infectiousness). The blue line shows when asymptomatic individuals are assumed to not be infectious at all.

From the figure above we notice that the greater shift in the distribution of cases when infectiousness of symptomatics is reduced occurs in the 1-15 year old age group. The reduction in the <1 year age group is not huge, presumably because transmission to this age group is from several sources as such reducing the infectiousness of symptomatics has little impact on the total numbers infected during an outbreak. We also notice that assuming asymptomatic cases are not infectious leads to far less number than were actually observed. This highlights the importance of asymptomatic individuals in transmission.

Following on from the simulation, we used a subset of the data that had only symptomatic episodes to re-estimate the model parameters (this is done to give an idea of how much information would be missed if the sampling had only been of individuals who showed symptoms). In this case, we still had days in the data with shedding but no symptoms and ARI episodes are not necessarily as long as the entire virus shedding episode.

Figure A. 24: Caterpillar plot of estimated parameters when only data from symptomatic episodes is used. The 15 parameters estimated and their respective effective sample sizes are shown. Points represent posterior medians, the thick lines represent 50% credible region and the thin lines represent 95% credible region. Except *η_A_* and *η_B_* (within household transmission coefficients) *ε_A_*, and *ε_B_* (community transmission coefficients) all the other parameters represent relative effects where a reference group exists. If a relative effect parameter is equal to 1(0 on the log scale) then the group it represents and the reference group are not different. Parameters where 50% credible interval overlaps with 0(dashed vertical line) are shown by open grey circles, where the 50% credible intervals do not overlap with 0 but the 95% credible interval does, filled grey circles show these parameters. If there is not overlap with 0, the circles are black and filled.


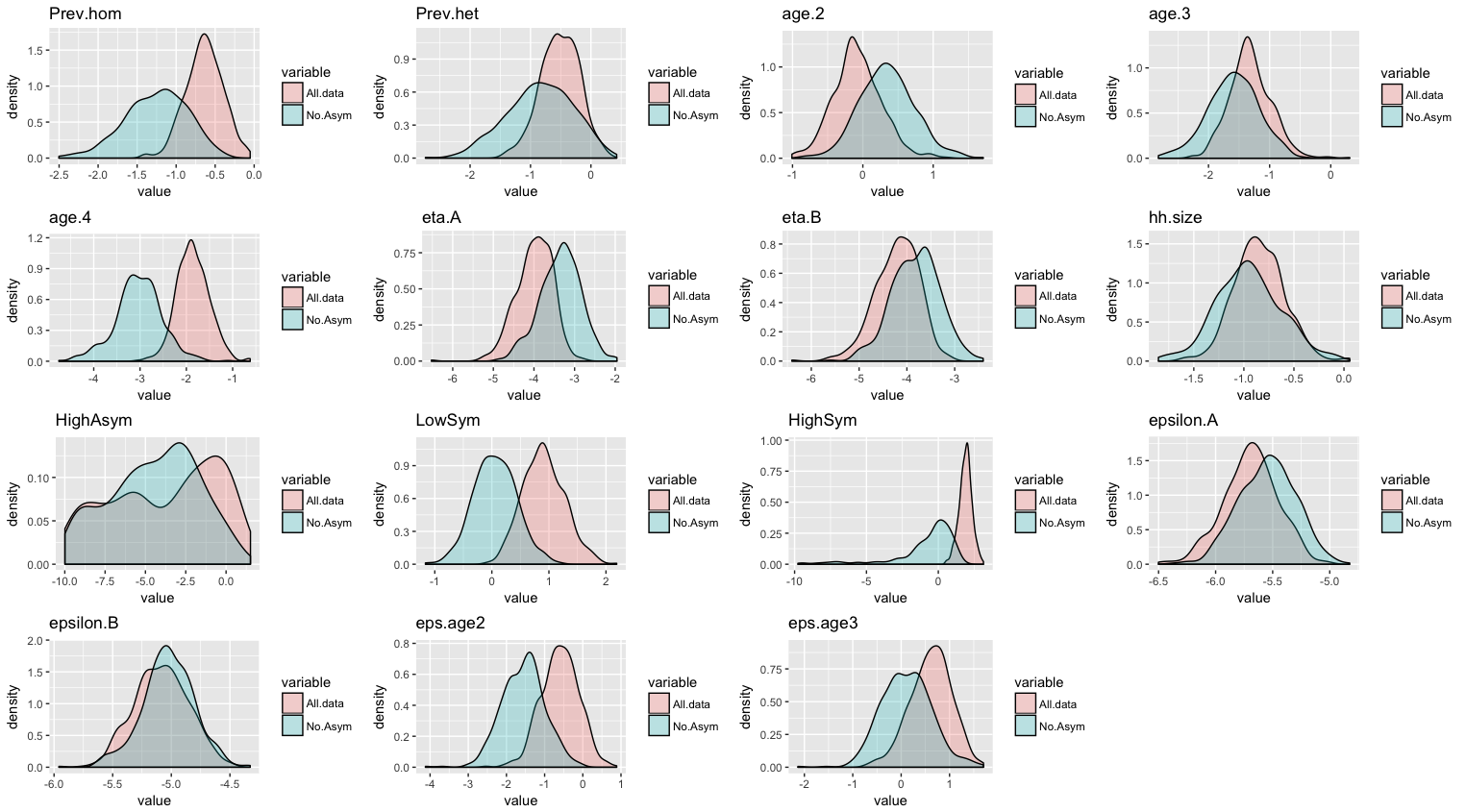


Figure A. 25: Comparing densities of parameters estimates obtained when using all the data (light red) to densities obtained when using data from only symptomatic cases (light blue).

# Fitting household size as an ordinal variable

As the model was built up in stages, this section was done prior to the inclusion of symptom data; instead only viral load was used as a proxy to infectivity. To fit household size as an ordinal variable, the rate of exposure equation is as below

$$\lambda_{ihg}\left( t \right)=exp(\phi_{X}X_{i}+\phi_{Yg}Y_{ig}(t))\left[ M_{ih}(t)\eta_{g}\left( N_{i}-1 \right)^{-\omega}\sum_{j\neq i} {\phi_{I}I}_{jhg}\left( t \right)+ \phi_{E}E_{i}\varepsilon_{g}f_{g}(t) \right]$$

The factor $\left( N_{ih}-1 \right)^{-\omega}$ modifies the within household transmission coefficient, where *N_i_* is the household size for susceptible *i* and ω determines that kind of transmission. If ω 🡪 0, it points to density dependent transmission, ω=1 implies frequency dependence. The estimation of ω was done using the entire data set and again using a subset where the definition of a household was changed such that a household is defined as individuals who share a building unit. The results of this are shown below.


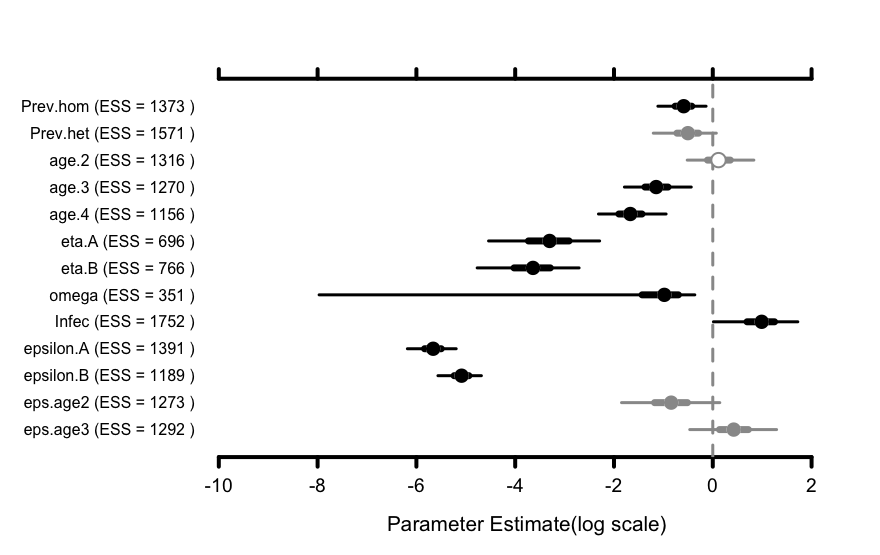


Figure A. 26: Caterpillar plot showing the results of estimating a parameter ω (omega) when household size is treated as an ordinal variable. These results were obtained when fitting was done using all the data available.


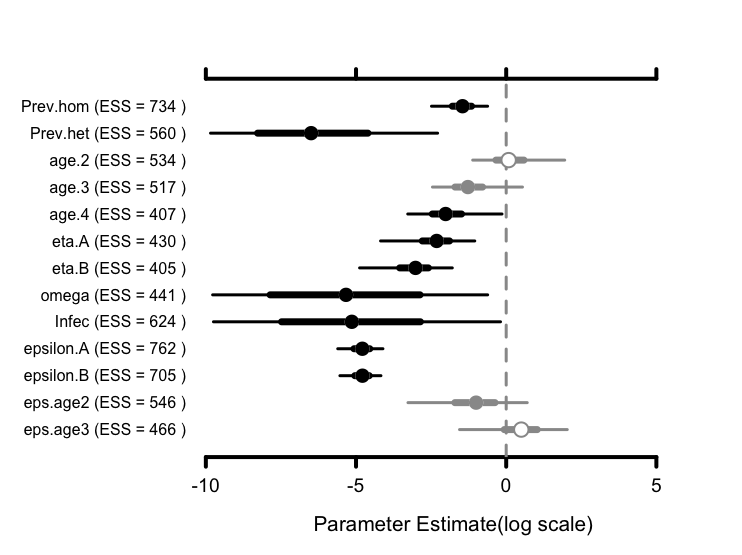


Figure A. 27: Caterpillar plot showing the results of estimating a parameter ω (omega) when household size is treated as an ordinal variable. These results were obtained when fitting was done using a subset of the data that had complete information on building units and hence a household could be redefined as a building unit.

Neither the entire data set nor the subset with redefined households seems to be able to give proper estimates of ω (omega). The distribution for this parameter is wide, but it should be noted that it does not include 1 (0 on the log scale) as such, the transmission is not frequency dependent in the usual notation. We also used the subset with redefined households to fit for a categorical effect of household size, the results of which are shown in Figure A. 28. The subset does not have enough information in it to narrow down on the effect of categorical household size, the effect of previous heterologous infection and the effect of high viral load. In fact, the latter distribution seems to have a reversed direction from previous results, implying high viral load reduces transmission. This is a curious result that perhaps further highlights the need to also use information on symptoms.


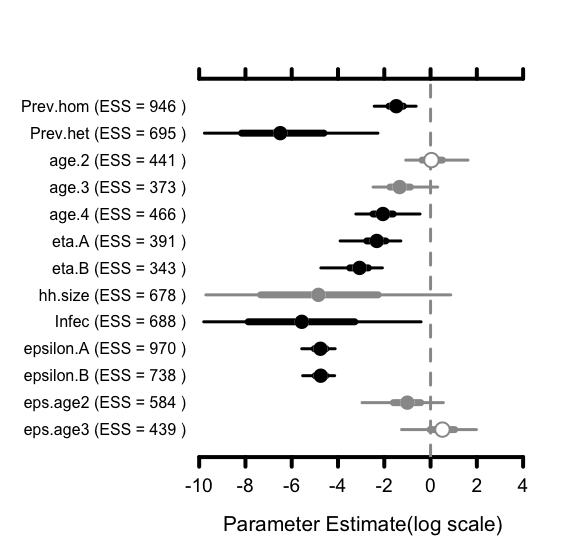


Figure A. 28: Caterpillar plot showing the results of estimation when household size is treated as a categorical variable but with the definition of a household changed. These results were obtained when fitting was done using a subset of the data that had complete information on building units and hence a household could be redefined as a building unit.

# References

Nolan, T., Hands, R.E., Bustin, S.A., 2006. Quantification of mRNA using real-time RT-PCR. Nat. Protoc. 1, 1559–1582.

Wathuo, M., Medley, G.F., Nokes, D.J., Munywoki, P.K., 2017. Quantification and determinants of the amount of respiratory syncytial virus (RSV) shed using real time PCR data from a longitudinal household study. Wellcome Open Res. 1, 27. https://doi.org/10.12688/wellcomeopenres.10284.2
